# Different Factors Affecting Human ANP Amyloid Aggregation and Their Implications in Congestive Heart Failure

**DOI:** 10.1371/journal.pone.0021870

**Published:** 2011-07-26

**Authors:** Lia Millucci, Eugenio Paccagnini, Lorenzo Ghezzi, Giulia Bernardini, Daniela Braconi, Marcella Laschi, Marco Consumi, Adriano Spreafico, Piero Tanganelli, Pietro Lupetti, Agnese Magnani, Annalisa Santucci

**Affiliations:** 1 Dipartimento di Biotecnologie, Università degli Studi di Siena, Siena, Italy; 2 Dipartimento di Biologia Evolutiva, Università degli Studi di Siena, Siena, Italy; 3 Dipartimento Farmaco Chimico Tecnologico, Università degli Studi di Siena, Siena, Italy; 4 Dipartimento di Medicina Clinica e Scienze Immunologiche, Università degli Studi di Siena, Siena, Italy; 5 Dipartimento di Patologia Umana e Oncologia, Università degli Studi di Siena, Siena, Italy; Université Joseph Fourier, France

## Abstract

**Aims:**

Atrial Natriuretic Peptide (ANP)-containing amyloid is frequently found in the elderly heart. No data exist regarding ANP aggregation process and its link to pathologies. Our aims were: i) to experimentally prove the presumptive association of Congestive Heart Failure (CHF) and Isolated Atrial Amyloidosis (IAA); ii) to characterize ANP aggregation, thereby elucidating IAA implication in the CHF pathogenesis.

**Methods and Results:**

A significant prevalence (85%) of IAA was immunohistochemically proven *ex vivo* in biopsies from CHF patients. We investigated *in vitro* (using Congo Red, Thioflavin T, SDS-PAGE, transmission electron microscopy, infrared spectroscopy) ANP fibrillogenesis, starting from α-ANP as well as the ability of dimeric β-ANP to promote amyloid formation. Different conditions were adopted, including those reproducing β-ANP prevalence in CHF. Our results defined the uncommon rapidity of α-ANP self-assembly at acidic pH supporting the hypothesis that such aggregates constitute the onset of a fibrillization process subsequently proceeding at physiological pH. Interestingly, CHF-like conditions induced the production of the most stable and time-resistant ANP fibrils suggesting that CHF affected people may be prone to develop IAA.

**Conclusions:**

We established a link between IAA and CHF by *ex vivo* examination and assessed that β-ANP is, *in vitro,* the seed of ANP fibrils. Our results indicate that β-ANP plays a crucial role in ANP amyloid deposition under physiopathological CHF conditions. Overall, our findings indicate that early IAA-related ANP deposition may occur in CHF and suggest that these latter patients should be monitored for the development of cardiac amyloidosis.

## Introduction

IAA is an age-related amyloidosis [1] limited to the atria and ANP is the major subunit of amyloid fibrils [2], [3]. ANP is synthesized by atrial cardiomyocytes [4], [5] and regulates the hydro-mineral homeostasis under normal and pathological conditions. Although its clinical relevance is unclear, patients with IAA are known to be prone to atrial fibrillation [6], [7]. Little is known about the putative role of IAA in the pathogenesis of cardiac arrhythmias in the elderly but a relationship between the incidence and the degree of IAA in cardiac disorders associated to high ANP plasma levels, and the incidence and the degree of IAA has been described, suggesting a prevalence of IAA in CHF [8]–[10]. Plasma ANP levels are elevated in CHF in relation to its severity [4], [11] as well as in IAA [12], [13], due to an increased ANP secretion by the failing heart.

Total circulating ANP is composed of three molecular forms [4]: α-ANP comprises 28 aminoacids with an intramolecular disulfide linkage; β-ANP (56 aminoacids), an antiparallel immer of α-ANP and γ–ANP (pro-ANP), composed of 126 aminoacids, carring the α-ANP sequence and stored in secretory granules of atrial cardiocytes. The circulating peptide exists also as a high MW form, as we showed [14]–[17], this latter not taking part in the polymerization process [17]. On the contrary, an ANP oligomeric circulating form can be the actual promoter of amyloid aggregation. Increased ANP concentration in severe CHF (NYHA class III and class IV) is due to the rise of β-ANP [4].

In IAA non-mutated ANP is the aggregating sequence [2], [3], but changes in the local environment or alteration of protein concentration are crucial for the onset of amyloidosis [18].

In this work, a significant prevalence of IAA was immunohistochemically proven in the atrial appendages of CHF patients showing an unequivocal relationship between these two pathologies. Moreover, we investigated for the first time ANP fibrillogenesis, starting from its monomeric form (α-ANP) as well as the ability of its dimeric form (β-ANP) to promote amyloid fibril formation. Different conditions were adopted, including those reproducing β-ANP prevalence in CHF [19]. Our results define the uncommon rapidity of α-ANP self-assembly at acidic pH supporting the hypothesis that these early developed aggregated forms constitute the onset of a fibrillization process subsequently proceeding at physiological pH. More interestingly, CHF-like conditions induced the production of the most stable and time-resistant ANP fibrils supporting the hypothesis that CHF affected people may be prone to develop IAA.

The study of the conformational preferences of ANP under different conditions appears to be crucial for shedding light on its intrinsic structural properties related to amyloid fibril formation. β-ANP can act as a seed for fibrillization in the heart and we suggest a role for β-ANP in amyloid deposition, discussing its pathophysiological significance in CHF and IAA.

## Results

### CR and anti-ANP immunohistochemistry

In the present stud we investigated the ANP tissue localization in the atrium of 40 CHF patients by means of CR assay and immunohistochemical analysis. Myocardial tissues (left atrial appendages) were obtained from patients with CHF that underwent surgery in the past 2 years (2009–2010). Biopsies stored in the tissues archives of “Patologia Umana e Oncologia” Department of the University of Siena were selected on the basis of pathologies and anonymously analyzed. For this reason in our Institution a specific written Informed Consent is not required. The detailed description of the whole study was presented and approved by local University Hospital Ethics Committee, the “Comitato Etico Locale dell'Azienda Ospedaliera Universitaria Senese”. Patients with diabetes mellitus and/or hypertension other than CHF were enrolled in the study. Table 1 summarizes concomitant pathologies, age and sex characterizing each patient who underwent biopsy. Light microscopy observations of the sections were made after hematoxylin-eosin staining and measurement of myocyte size (mean diameter 20–40 myocytes/specimens); the degree of IAA deposition in the atrial wall was graded subjectively on a scale of 0–3 and the highest grade from the sites evaluated was taken as representative for each heart. Paraffin sections were also stained with CR and immunostained with the antibody to ANP as shown in Figure S11.

**Table 1:** Patients Characteristics. Values are means±SD or n (%).

Features	Patients	CR Negative	CR positive
**Total**	40	2(5)	38(95)
**Age, years**	59.4±9.3	49.5±1.5	61.2±8.7
**Sex (M/F)**	22/18	2/0	20/18
**Disease**			
** CHF**	40(100)	2(100)	38(100)
** Hypertension**	31(77.5)	1(50)	30(79)
** Diabetes mellitus**	14(35)	2(100)	12(32)
**Immunohistochemistry**			
** ANP negative**	6(15)	2(100)	4(10.5)
** ANP positive**	34(85)	0(0)	34(89.5)

In patients with amyloidosis the myocytes size was larger (21.1 µM ±2) than usually reported in normal hearts (∼13 µM) [20] and amyloid was found in 38 (95%) of 40 patients; grade 2–3 IAA was demonstrated in 34 CHF hearts (85% incidence). The association between IAA and CHF was independent of age and sex, anyway our data revealed a good correlation between ANP amyloid deposition and CHF patients conditions.

### Kinetics of α-ANP aggregation

#### pH 7.4

Monomeric α**-**ANP in H_2_O pH 7.4 at growing concentrations was incubated at 25°C and analyzed for CR binding (Cb) as shown Figure S1 and Figure S8 and a slow concentration-dependent increase in absorbance was observed. Aggregation exhibited a threshold-type behaviour where rates increased once 10 µM α-ANP was reached, which was adopted as the reference concentration. This trend was confirmed by Th-T assay as shown in Figure S2.

#### pH 4.0

**Figure 1:**
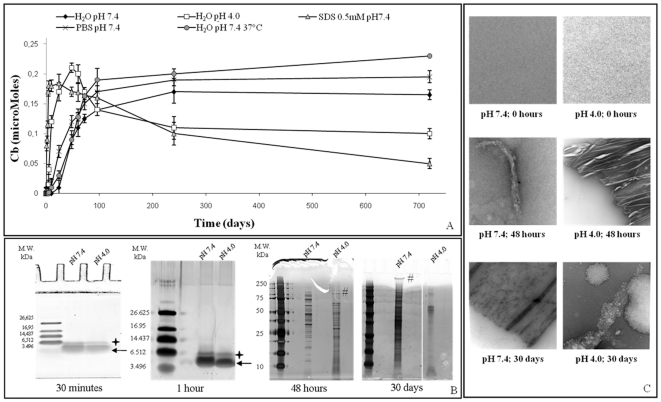
Aggregation kinetics. **A**) ***Aggregation kinetics of α-ANP via Congo red binding method.*** Data represent the mean of at least three independent experiments (n≥3). Errors bars represent standard deviations **B**) ***Glutaraldehyde Cross-linking, Tris-tricine SDS-PAGE and SDS-PAGE at different times***. The formation of α-ANP oligomers and high MW aggregates was monitored. Asterisks indicate dimers, arrows indicate monomers and # indicate high molecular weight aggregates. **C**) ***Morphology of α-ANP aggregates at different times and in different conditions.*** α-ANP was applied onto a copper grid and negatively stained with 2 (w/v)% uranyl acetate. Scale bar:100 nm.

By incubating α-ANP in H_2_O at pH 4.0 a strong decrease of the lag phase in comparison to pH 7.4 was evident as shown in Fig. 1A and this find was in keeping with Th-T experiments observable in Figure S3. Moreover, at pH 4.0, Cb values decreased starting after 50 hours, indicating a change in α-ANP fibrillar state.

### Electrophoretic patterns of α-ANP aggregation

Tricine SDS-PAGE using sample glutaraldheyde cross-linking was adopted for the detection of stable α-ANP multimers formed during the first hour of incubation as shown in Fig. 1B. Figure S4 shows that α-ANP at pH 4.0 and 7.4 spontaneously associated into immer_ in a few minutes. α-ANP incubated at pH 4.0 produced oligomers growing in size more rapidly than at physiologic pH, but at the final aggregation state, only pH 7.4 solution showed heavy aggregates in the upper gel and a consistent oligomers amount as shown in Fig. 1B, suggesting a peculiar thermodynamic equilibrium between fibrils and oligomers. This accounts for the immer being the most stable complex at physiologic pH and indicates that formation of higher order oligomers was barely due to interactions of the immer with peptide molecules in its surrounding. Fig. 1C shows α-ANP morphologies at different aggregation times.

### Factors influencing the kinetics of α-ANP aggregation at pH 7.4

We studied the starting solvent temperature and ionic strength effects on α-ANP aggregation kinetics as shown in Fig. 1A. Fibrils formation was promoted in PBS suggesting that aggregation was driven by a hydrophobic effect, salt bridges playing a role in fibrils' stabilization, thus forming nuclei rapidly [21]. These results were confirmed by Th-T assay as shown in Figure S3. α-ANP aggregation was also dependent upon temperature showing an appreciable shortened lag phase if incubated at 37°C. Finally, we examined the aggregation in the presence of SDS [22] founding that 0.5 mM SDS, close to 0.75 mM critical micelle concentration (CMC) [23], induced the fast α-ANP fibrils growth at physiological pH shown in Fig. 1A, probably through the SDS-induced conformational change of α-ANP monomers.

### Infrared analysis of ANP aggregation (see Text S1)

**Figure 2:**
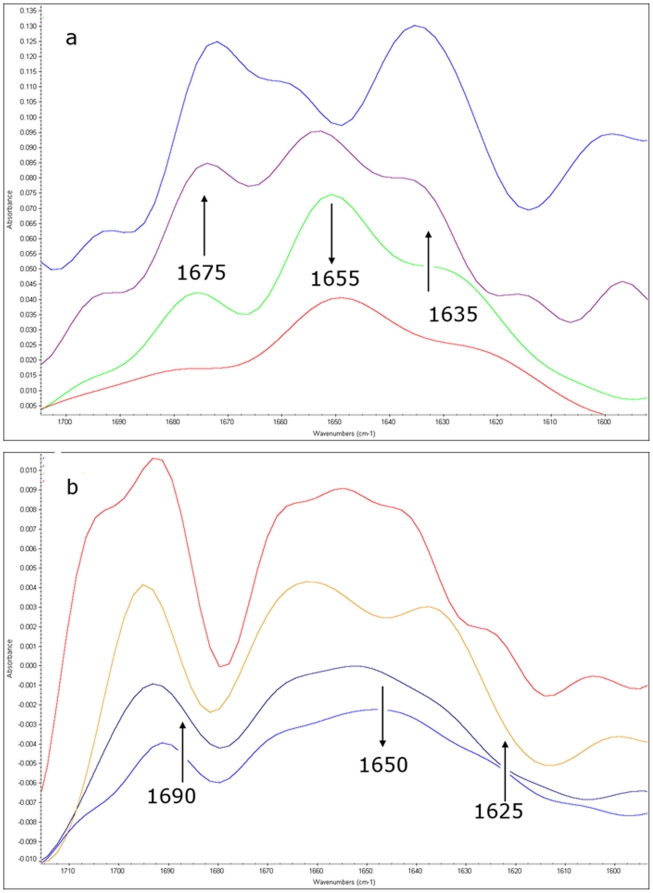
Fourier-self deconvoluted Amide I spectral region of α-ANP in aqueous solution. **a:** pH 7.4, from the bottom to the top t = 0 h, t = 5 h, t = 30 h, t = 168 h. **b:** pH = 4.0, from the bottom to the top t = 0 h, t = 1 h, t = 48 h, t = 168 h. Arrows indicate the increase/decrease of the relative band intensities with time.

The representative Fourier-self deconvoluted Amide I spectral region of α-ANP in aqueous solution, collected at different times under two different pH conditions (7.4 and 4.0), are shown in Fig. 2 and in Figures S9 and S10. At pH 7.4 two shoulders at 1635 and 1675 cm^−1^, attributed to the packing of β strands into β-sheets and to the subsequent formation of intermolecular β-sheet aggregates, reached the maximum intensity (then remaining constant) after 7 days, revealing that the formation of these aggregates occurs with a very slow kinetics and giving stable aggregates, whereas the aqueous solution was converted into a gel-like system.

At pH 4.0 the 1625 and 1685 cm^−1^ contributions of the Amide I band, attributed to β-sheet structures and intermolecular β-sheet aggregates, immediately appeared in the spectrum of α-ANP shown in Fig. 2b and their intensity increases quickly, suggesting a very rapid aggregation kinetics. The α-ANP IR spectra recorded from 48 to 168 hours were not any more superimposable indicating that the aggregated structures are not so stable under these conditions, according to the microscopic observation. Moreover, the α-ANP solution after 7 days looks like an aqueous solution and not as the gel-like system observed at pH 7.4.

### α-ANP preformed aggregates seeding ability

**Figure 3:**
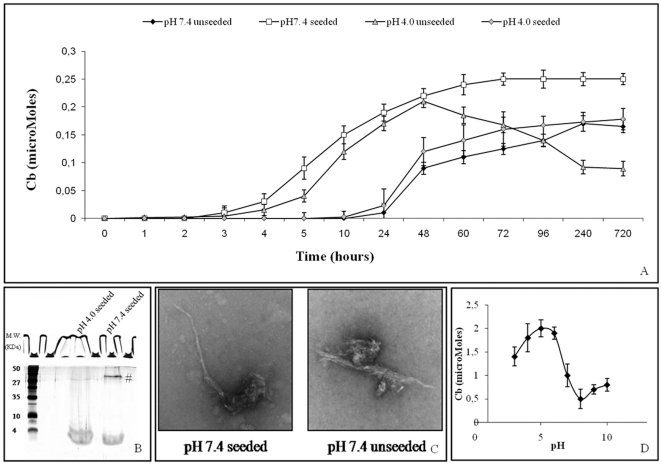
Seeding. **A**) ***Seed-induced fibril formation of α-ANP*** **.** Seeds were generated from aged α-ANP (pH 7.4 and pH 4.0, respectively) and added as percentage weight fractions. Fibril formation was monitored over time by Cb and TEM. **B**) ***Treatment in 90% HFIP of ANP aggregates formed after seeding*** **.** High MW aggregates from pH 4.0 seeded solution remained on the top of the gel while aggregates formed by pH 7.4 seeds were monomerized. **C**) ***Morphology of samples aged for 720 hours.*** Morphologies of seeded and unseeded α-ANP at pH 7.4 were still similar in shape. **D**) ***pH dependence of α-ANP aggregation.*** α-ANP was incubated at various pH and after two days the amount of aggregation was quantified by Cb.

We compared the abilities of α-ANP aggregates preformed at pH 7.4 and pH 4.0 to act as seeds for initiation of fibril formation and Fig. 3A illustrates the results of the performed experiments. Aggregation was measured by Cb and Th-T assays. α-ANP aggregates preformed at pH 4.0 were able to stimulate fibril formation to achieve a complete aggregation and to induce a propagation of amyloid structure at pH 7.4. In contrast, aggregates preformed at pH 7.4 were unable to accelerate fibril formation when added to the pH 4.0 solution. Incubation of α-ANP at pH 4.0 in the presence or absence of seeds resulted in a rapid high-yield formation of amyloid fibrils as shown in Fig. 3A. TEM images of α-ANP aggregates formed at physiologic pH from pH 4.0 seeds showed a typical, well defined amyloid morphology, analogous to the fibrils prepared at pH 7.4 as shown in Fig. 3C. These results indicate that, following a pH shift from 4.0 to 7.4, the acidic pre-aggregates adapted to the physiologic pH. Such aggregates were also remarkably stable: high MW aggregates remained after treatment with 90% HFIP, drying and resuspension in boiling sample buffer, while aggregates formed at ph 4.0 from aggregates preformed at pH 7.4 were monomerized as shown in Fig. 3B. These findings suggest that: i) the stability of α-ANP amyloid fibrils is optimal at physiologic pH; ii) the presence of moderate concentrations (4%) of pH 4.0 pre-formed seeds may be necessary to partially destabilize α-ANP monomer and make it amenable to rapidly form stable and resistant aggregates. As shown in Fig. 3D, such results are supported by the observation that the extension of α-ANP amyloid fibrils were greatly dependent on the reaction mixture pH, with an optimum around 4.0–6.0.

#### CHF α/β-ANP ratio

**Figure 4:**
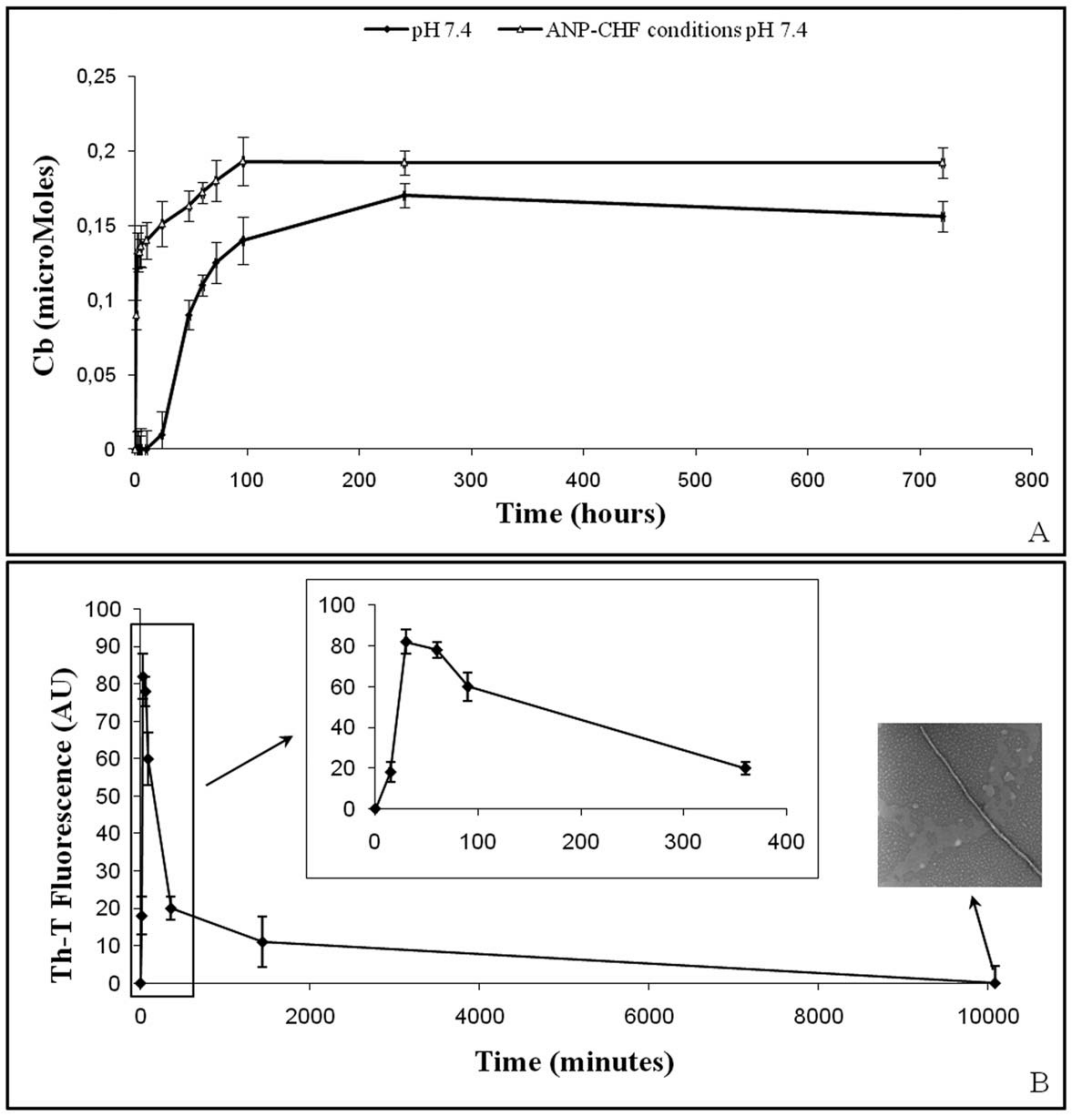
Time course of ANP-CHF fibril formation. **A**) Cb and **B**) Th-T fluorescence**.** TEM image after 30 days showed amyloid fibril larger in cross sectional area and longer in length than fibrils formed in all other conditions tested.

To simulate α/β-ANP ratio occurring *in vivo* in CHF patients (ANP-CHF), experiments were performed starting aggregation with a mixture of 10 µM ANP immer_ (β) and monomers (α), ratio 2∶1, as shown in Figure S7. The rate of Cb-monitored aggregation of α-ANP at pH 7.4 was significantly enhanced by seeding slow aggregating solution with β-ANP as shown in Fig. 4A and as confirmed by Th-T experiments illustrated in Fig. 4B. Interestingly, Th-T and Cb kinetics didn't correlate. The maximum value of fluorescence was followed by a very rapid decrease which was not consistent with aggregation signals measured by Cb. When analysed by TEM, final aggregates showed assembled fibrils (Fig. 4B). This finding well correlates with substantial increase of aggregates' levels that gave CR red shift and shoulder peak at 543 nm, even if these structures did not generate Th-T fluorescence.

### Fibril morphology

#### ANP-CHF

**Figure 5:**
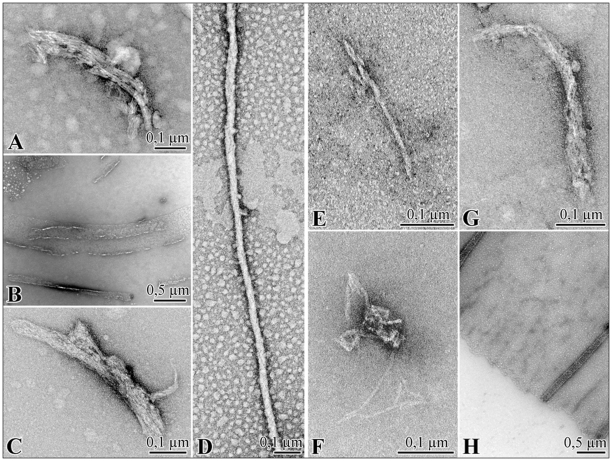
TEM images. Electron micrographs showing the ANP-CHF time course aggregation at pH 7.4 (**A,B,C,D**) and at pH 7.4 (**E,F,G,H**).

*In situ* generation of ANP-CHF aggregates on grids at pH 7.4 did not influence morphology (data not shown). At time 0, oligomers on grid had granular structure, at 45 minutes, ANP-CHF aggregates exhibited the standard features of amyloid fibrils showing a substructure composed of parallel alignments of smaller filaments visible in Fig. 5A. Aggregates at 120 minutes included thin fibrils with a faintly discernable periodicity, planar aggregates, approximately 10±2.56 nm thick visible in Figure S5. At 48 hours, fibrillar precipitates formed with widespread aggregations arranged in lateral arrays of fibres often superimposed as shown in Fig. 5C. An increase in size of aggregates was observed and unordered and flat assembly structures persisted following aggregation to cover fibrils bundles uniforming the surface area as shown in Fig. 5B. At 48 h fibrils were entangled, highly superimposed and bundled. Figure S5 shows a sample after 3 weeks of incubation and individual strand diameters within the bundles ranged in diameter 3–8 nm; after 1 month sample consisted in long, straight, matures fibres while other kinds of aggregates were completely disappeared as shown in Fig. 5D. Fibrils had approximately 20 nm diameter and indeterminate length, with a twist in their longitudinal axis. Once formed, aggregates were very stable (see below). Together, these data suggest that an overabundance of immer_, corresponding to severe CHF conditions, may enhance ANP fibrillogenesis promoting amyloid aggregation.

#### α-ANP at pH 7.4

Light microscopic examination of α-ANP assemblies revealed that both thin, sheet-like structures and fibrous aggregates were formed as shown in Figure S5. Ultra-structures were examined electron microscopically. At starting incubation time, α-ANP contained neither fibrils nor sheet-like clusters confirming the monomeric nature of peptide as shown in Fig. 1C. After 1 hour, α-ANP formed irregular, lumpy, occasionally branching protofibrils 10–100 nm long visible in Fig. 5E and after 24 h, 200–400 nm long smooth, straight fibrils visible in Fig. 5F. At 48 hours, small, irregular protofibrils and strictly interconnected, fibrillar macroaggregates were present, while after 6 days, some mature amyloid bent and entangled strands were observed as shown in Fig. 5G. After 30 days α-ANP showed precipitates bearing extensive aggregates, arranged in lateral arrays of fibrils assuming a planar conformation, with ribbon-like fibres strictly assembled and stratified which peculiar morphology is well appreciable in Fig. 5H.

#### α-ANP at pH 4.0

**Figure 6:**
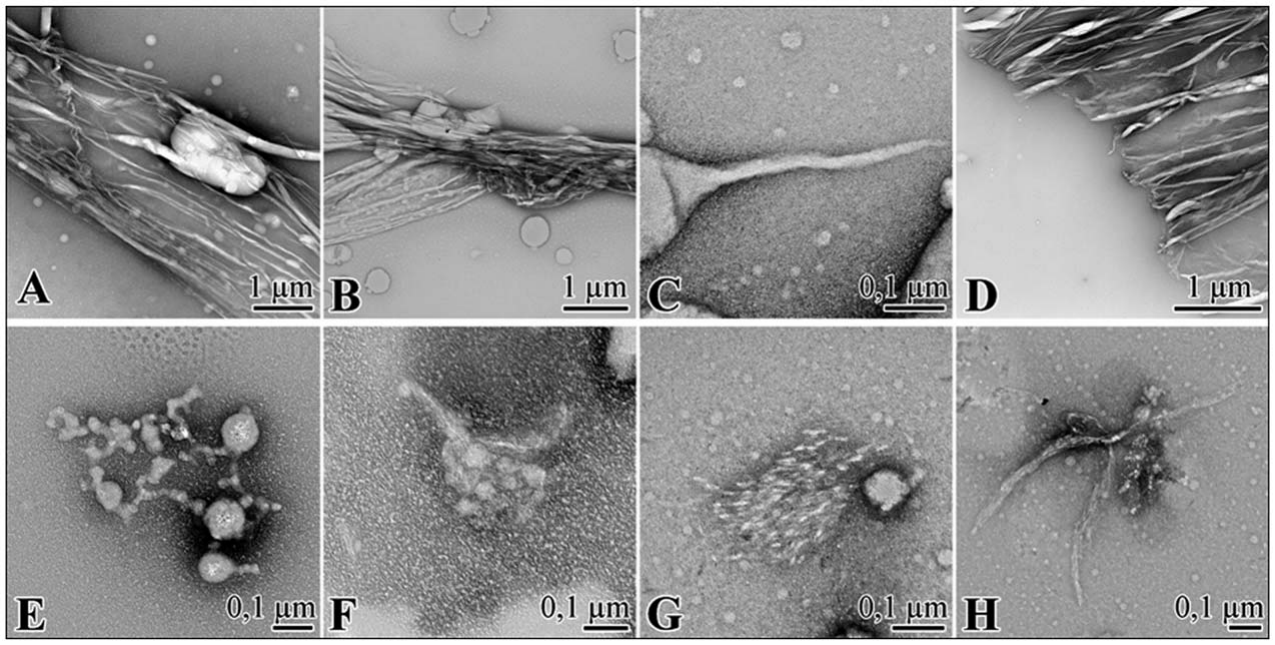
TEM images. Electron micrographs showing the α-ANP time course aggregation at pH 4.0 (**A,B,C,D**) and in 0.5 mM SDS at pH 7.4 (**E,F,G,H**).

At pH 4.0 α-ANP spontaneously and rapidly assembled to form amyloid-like fibrils with peculiar morphologies. Figure S5 C shows that after 90 minutes incubation ANP formed wide flakes with a diameter of 20–30 nm. After 4–7 h globular structures were still observed in concomitance with early fibrillar structures shown in Fig 6C. At 24 hours, the supramolecular spherical structures were formed and were larger than most previous reports of soluble oligomers, ranging from a few nm to 1 µm or more in diameter visible in Fig. 6A. Surprisingly, many of them appeared to be interconnected with fibrillar material suggesting a direct association between the two morphological species and a potential intermediary role for the large peptide assemblies as shown in Figure S5. Fig. 6D shows that after 48 hours mature, well-ordered strands 2–3 µm long and 10–40 nm wide were formed. These fibrils were long, straight and twisted, clearly indicative of a helical arrangement of protofilaments. We also observed many twisting ribbon-like structures of different width and pitch and flat ribbons of parallel protofilaments.

#### α-ANP in 0.5 mM SDS

The addition of 0.5 mM SDS promoted the formation of α-ANP fibrils avoiding the lag time as observed by TEM. At the starting incubation time, reaction mixture was composed by a sparse number of amorphous clusters and large spherical structures as shown in Fig. 6E. 2 hours later, sample exhibited numerous tiny globules and slight, linear fibrous structures shown in Fig. 6F, evolving in some thin protofibrillar elements extruding directly from globules, giving rise to interweaved aggregates. After 6 hours a net change in morphology was observed, the clusters developing into more extensive ordered lattice-like structures and showing several protofibrillar elements orbiting the SDS-micelles. At 30 hours, globular structures were always present with bundles of strictly assembled short fibrils, formed through coalescence and progressive fibrils addition around micellar structures as shown in Fig. 6G. Fig. 6H shows some amorphous aggregates, in concomitance with rigid bifurcate fibrillar material the were also present in the solution (Fig. 6H). This aggregated material was not stable, since further incubation only produced amorphous, globular particles without fibrils.

### Stability of ANP amyloid aggregates

**Figure 7:**
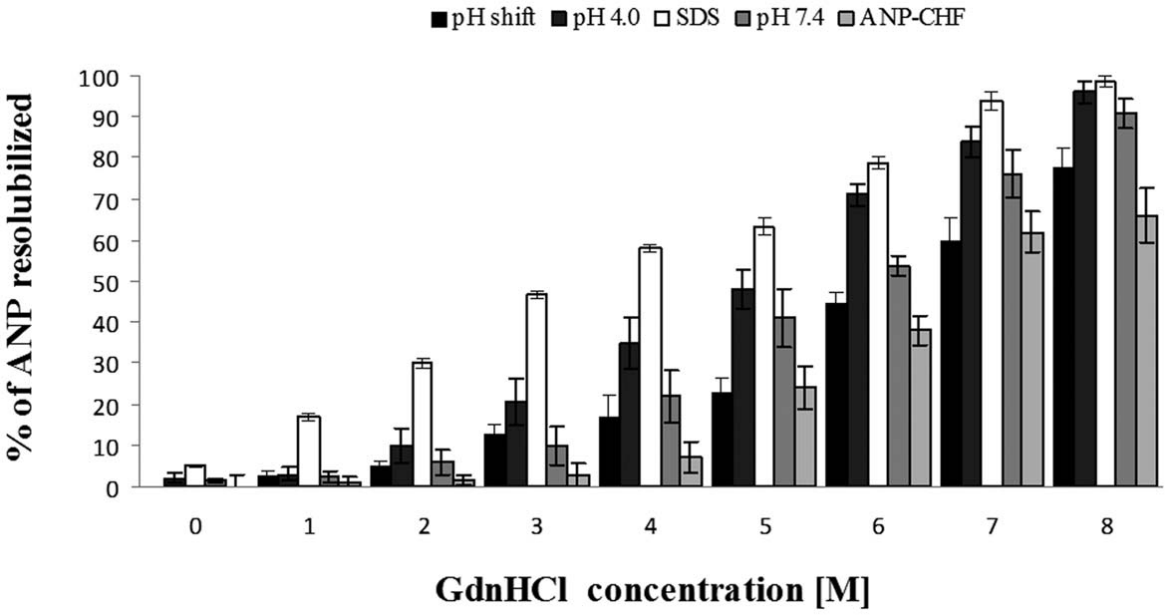
Stability of ANP aggregates *via* GdnHCl addition. ANP-CHF fibrils were the most resistant since only above 4 M GdnHCl, an increasingly greater percent of total fibrils was resolubilized.

The addition of DTT reduced amyloid fibril formation in a dose-dependent manner as shown in Figure S6 at both 7.4 and 4.0 pH. This effect was common to Cys-containing peptides, suggesting that the prevention of oxidative dimerization by the disulfide bond may retard fibril formation. Moreover, to evaluate the intrinsic stability of aggregates generated under different conditions, we examined the fraction of denatured fibres as a function of GdnHCl concentration and we illustrate the results in Fig. 7. Fibrils formed by ANP-CHF in 0–4 M GdnHCl were only minimally dissolved. Above 4 M GdnHCl, fibrils were dissolved to a greater extent up to 8 M GdnHCl, where most of them were dissolved. Fibrils formed at pH 7.4 by α-ANP reflected the ANP-CHF stability trend, although at a diminished extent. Conversely, fibres formed by α-ANP at pH 4.0 were already partially dissolved at 4 M GdnHCl and mostly dissolved at 6 M GdnHCl. Finally, fibres formed in 0.5 mM SDS were extensively dissolved with 4 M GdnHCl, confirming to be the less stable. Surprisingly, α-ANP fibres formed at pH 4.0 and then shifted at pH 7.4 ,“pH shift”, reached values comparable to ANP-CHF and were only partially dissolved in 4 M GdnHCl, demonstrating that pH shift contributed to stabilize and strengthen fibre's structure. This suggests that the maturation of fibrils is an important step of IAA molecular mechanism.

## Discussion

In the present paper, we provided the first experimental evidence of a close correlation between CHF and IAA revealing the prevalence of IAA in CHF patients. Moreover, experimentally we proved that β-ANP, the major circulating ANP form in CHF [4], [7], [11], is *in vitro,* the seed of ANP amyloid aggregation yielding extremely stable and resistant fibres. It is commonly assumed that extensive intra-atrial amyloid deposition occurs only after 60 years of age and because of unknown factors [1], [2], [10], [13]. Our findings suggest an alternative possibility, *i.e.* that the formation and maturation of amyloid fibrils may start in the early stages of cardiac failure when the pathophysiological milieu provides a suitable substrate and the balance between formation and proteolytic degradation shifts towards favouring amyloid deposition. First stage fibrils formed soon after the start of cardiac disease might be unstable, requiring additional stabilizing factors such as preformed seeds or membrane association.

ANP plasma levels closely parallel the instantaneous secretion rate, and therefore likely they greatly vary in response to different pathophysiological stimuli or according to peculiar pathologies. β-ANP, having a diminished cGMP generating potency, is the main form of circulating ANP in CHF patients [8], [11].

In the atrium, where ANP is secreted, β-ANP may accumulate and easily aggregate. The discovery that amyloid-positive substance is strikingly associated with atrial myofibrils in human hearts with dilated or hypertrophic cardiomyopathies [13], suggests IAA as a potential major pathogenic process in CHF. Changes in energy production, energy utilization and excitation-contraction coupling are reported in heart failure [9] and such changes in cardiac metabolism may be involved in the augmentation of β-ANP synthesis. Processing of ANP precursor in the human failing heart differs from that in the normal heart. Our findings on acidic pH seeds accelerating and stabilizing ANP fibril formation and on SDS micelles contact enhancing aggregation, together with the observation of IAA fibrils are also present inside atrial myocytes [10], [13], strongly validate the importance of initial solution conditions affecting ANP amyloid aggregation and stability, playing β-ANP the main effective role in producing the most quickly assembled and strong fibrils. The amyloid state of endocrine hormones in secretory granules of the heart atrium contrasts the historical disease association of amyloids in other organs. On the other hand, ANP may not be very toxic because the hormone amyloid is stored inside the granules and its aggregation and secretion may be highly regulated, including by means of pro-hormone processing. If ANP homeostasis is altered under stress, age or CHF conditions, hormone aggregation may be out of control and disease-associated amyloid aggregation may occur. Whether such aggregation causes disease, or is an indirect effect of the protein homeostasis altered by disease, remains undetermined. In fact, pro-hormone maturation seems mostly a feature of atrial peptide synthesis and enzymes involved in pro-ANP maturation are produced predominantly in atrial myocytes [24], while ventricular myocytes don't contain secretory granules for peptide storage and maturation. Pro-ANP post-translational processing may in CHF lead to β-ANP production. In agreement, CHF patients have increased β-ANP plasma concentrations [4], and CHF may not be ameliorated by secreted ANP [25].

Although the mechanism of the initial nucleation step in patients remains unknown, the maturation of fibrils via acidic pH-seeding is likely to be involved in the development of IAA amyloidosis. Since the levels of circulating ANP are relatively low [26], it is unclear where and under which conditions fibril initiation might occur *in vivo*. One possibility, analogous to other amyloid systems, is that fibril formation might be initiated in the low pH environment of the secretory granules before continuing in a higher pH environment. This hypothesis is supported by our findings that optimal pH range for ANP aggregation is 4.0 to 6.0 and that i*n vitro* ANP-aggregates form rapidly upon incubation at pH 4.0. Our work proved, for the first time, how different incubation conditions greatly affect not only aggregation kinetics but also ANP fibril morphology. Although the participation of additional factors stabilizing the amyloid fibrils is possible, our findings define that, by acid pH-seeding, ANP amyloid fibrils adapt to neutral pH conditions and transform into stable matured fibrils. Acidic pH seeding causes the fibrils to mature and the amount of fibrils to increase and become stable without additional factors, finally resulting in a massive amyloid accumulation. These results are consistent with the hypothesis whereby aggregation may be initiated intracellularly and end up as a pathological hallmark in the extracellular space.

**Figure 8:**
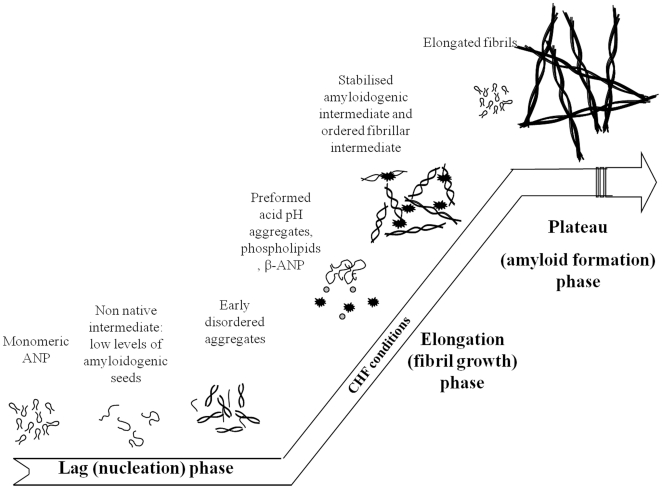
Theoretical schema of ANP aggregation.

The observed acceleration of ANP fibrils formation can be described by the scheme in Fig. 8 that hypothesizes the existence of two types of fibrils: i) fibrils formed after maturation in the cytosol of seeds produced at acid pH, and ii) a second kind concerning the peculiar CHF conditions, where the β-ANP immer is the most prevalent circulating form.

Although the information resulting from our *in vitro* experiments provide an insight into ANP aggregation process, a comparison of ANP *ex vivo* and *in vitro* fibrils is still required, to directly relate our findings to *in vivo* fibrillogenesis and to conclusively account for the involvement of circulating β-ANP massively produced in CHF in ANP amyloid deposition. Unfortunately, the retrieval of sufficient amounts of fresh and correctly collected human CHF endomyocardial bioptic material for TEM analysis is still a hard problem on which we are currently focusing for further studies.

Our findings suggest that, since IAA may occur at a younger age than its usual post-mortem diagnosis and early IAA-related ANP deposition may occur in CHF. Patients with a diagnosed CHF should be examined histologically, because ANP amyloid infiltrations may be found also in patients with no clinical symptoms and young patients having evidence of CHF will almost always develop IAA. Actuarial study confirms the significant adverse influence of cardiac involvement in ANP amyloidosis.

## Materials and Methods (see Text S1)

### Congo Red (CR) staining and anti-ANP immunohistochemistry

Atrial biopsies from 40 CHF patients listed in Table 1 were stained with CR for light microscopic evaluation. ANP was identified as CR–positive amyloid by immunohistochemistry.

### Ethics Statement

The whole study was conducted following the approval of the local University Hospital Ethics Committee, namely “Comitato Etico Locale dell'Azienda Ospedaliera Universitaria Senese”. The investigation conformed with the principles outlined in the Declaration of Helsinki.

Myocardial tissues were obtained from biopsies stored in the tissues archive of “Patologia Umana e Oncologia Department” of the University of Siena; appropriated samples were selected on the basis of pathologies and anonymously analyzed. For this reason in our Institution a written Informed Consent is not required. The detailed description of the whole study was presented and approved by the “Comitato Etico Locale dell'Azienda Ospedaliera Universitaria Senese”.

### Aggregation kinetic measurements

ANP: synthetic ANP (AnaSpec Inc), after treatment with 1,1,1,3,3,3-hexafluoro-2-propanol (HFIP, Sigma), was resuspended to the desired concentration in the appropriate buffer. Aggregation kinetic parameters were obtained by monitoring the reaction by Congo Red binding (Cb) method [5] ^.^ and Thioflavin –T (Th-T) fluorescence [27], [28]. Reactions were initiated by incubating appropriate concentrations of freshly prepared α-ANP in the desired buffer.

### ANP-CHF preparation

ANP (1 mg/mL ) was dissolved in H_2_O pH 7.4 at and centrifuged at 16,500× *g* for 1 h. The supernatant was collected and filtered with a 20 nm pore filter. Protein concentration was determined at 280 nm using [29].

### Electrophoresis

ANP **s**amples incubated for 1 hour were analyzed by glutaraldehyde cross-linking [30] and analyzed by SDS-PAGE or Tricine SDS-PAGE.

### FTIR measurements

The spectra were obtained with a Thermo Nicolet 5700 Fourier Transform Infrared spectrometer operating between 3000 and 900 cm^−1^ and 100 scans at a resolution of 2.0 cm^−1^ were averaged. An ATR (Attenuated total reflectance) cell equipped with a 45° germanium IRE (internal reflection element) crystal was used to record the water spectra and ANP solution. The spectra were taken in a single beam mode at predetermined time intervals to obtain kinetic information of the peptide aggregation process. To improve the observability of the overlapping bands, mathematical resolution enhancement was performed by a spectral deconvolution process.

### Cross-seeding experiments

Fibrils were sonicated and then incubated at ph 4.0 and pH 7.4 to be used as seeds in cross-seeding experiments.

### Disaggregation assay

Aliquots of mature ANP fibrils suspensions formed under different conditions were resuspended in guanidine hydrochloride (GdnHCl) at growing concentrations (0–8 M) and protein concentration of obtained monomers/oligomers was determined [31], [32].

### Transmission Electron Microscopy

Samples were suspended in deionized water, diluted 100-fold and then placed on a glow-discharged 200 mesh carbon coated copper grid. After to be adsorbed and air dried samples grids were then stained with 2% uranyl acetate for 45 s. Excess stain was removed, and the samples were allowed to air-dry and analyzed utilizing a Philips CM10 TEM operating at 80 kV.

## Supporting Information

Figure S1 **Aggregation kinetics** ***via*** **Congo Red.** Time course of α-ANP aggregate formation using α-ANP previously disaggregated by treatment with HFIP (see Methods) and incubated at different concentrations at pH 7.4. Aggregate formation was measured by Congo Red binding method. Arrow indicate the magnification of red box area.
(PPT)

Figure S2 **Aggregation kinetics** ***via*** **Th-T.** α-ANP amyloid aggregation monitored by Th-T fluorescence. α-ANP HFIP treated was incubated at different concentration at pH 7.4. Arrow indicate the magnification of red box area.
(PPT)

Figure S3 **Aggregation kinetics of α-ANP** **in different conditions.** **A**) Effect of pH, solvent type and temperature on the aggregation kinetics of α-ANP measured *via* Th-T fluorescence. **B**) Seed-induced fibril formation of ANP in H2O (pH 7.4) at 25°C. The seeds or pre-existing fibrils were generated from aged ANP (pH 7.4 and pH 4.0 respectively) and were added as percentage weight fractions. Fibril formation was monitored over time by Th-T fluorescence. **C**) pH dependence of α-ANP aggregation. α-ANP at 0.5 mg/ml was incubated at 25°C at various pH values and after two days the amount of aggregation quantified by Th-T fluorescence.
(PPT)

Figure S4 **Formation of α-ANP oligomers and aggregates monitored by Tris-tricine SDS-PAGE.** **A**) α-ANP solution in H2O at pH 7.4 at different aggregation times; **B**) α-ANP solution in H_2_O at pH 4.0 at different aggregation times; an appreciable difference in oligomers size and distribution between the two tested solutions was evident. Arrow indicates monomer, asterisk indicates dimer and # indicates high molecular aggregates.
(PPT)

Figure S5 **Microscope observations.** **A**) CHF-ANP incubated at pH 7.4; Images show branched fibrils indicating that they are composed of interwined protofibrils and aggregates composed of fibrils with apparent different morphology. **B**) TEM micrographs of α-ANP incubated at pH 7.4: images show several fibrils that lie almost parallel and close to one another. One or more protofilaments unwinding from a fibril and winding onto an adjacent one are also observed. On the right, light microscopy of the α-ANP assembly is shown; CR brightfield (Magnification 20X); **C**) TEM observations of α-ANP incubated at pH 4.0 showing loose aggregates in which individual fibrils can be seen. Bundles of fibrils diverging from spherulites are evident.
(PPT)

Figure S6 **Effect of DTT addition on α-ANP incubated at pH 7.4 and pH 4.0.** DTT is able to dose-dependently reduce the amount of amyloid fibrils formed under both pH 7.4 and pH 4.0 conditions.
(PPT)

Figure S7 **Electrophoresis.** To model the conditions occurring in vivo in the heart of CHF patients, a mixture of ANP dimers and monomers (ratio 2∶1) roughly as in CHF conditions, was settled up. Figure shows Tricine SDS PAGE of ANP in H2O pH 7.4. 1) Untreated sample; 2) Treated sample. Quali-quantitative image analysis was performed on silver stained gel by using Image Quant (Master) software to assess the obtaining of monomer/dimer 1α-ANP:2β-ANP ratio as shown in the Table.
(PPT)

Figure S8 **CR assay. A**) Spectral features of CR and aggregated α-ANP. Absorbance spectra of a suspension of α-ANP in the absence and the presence of CR and of CR alone; **B**) When CR binds to excess fibrillar α-ANP, a change in colour from orange–red to rose is induced that corresponds to a shift in the characteristic absorbance spectrum of CR. **C**) Relationship between the shoulder peak (543 nm) and time, for 10 µM α-ANP in 20 µM CR. The original absorbance spectra at 541 nm at interval of 30 seconds on the aggregation of α-ANP are shown. The shoulder peaks at 541 nm gradually grew with time, indicating that CR is kinetically bound to the amyloid aggregates.
(PPT)

Figure S9 **Representative infrared spectra of α-ANP in aqueous solution at pH = 7.4.** Representative infrared spectra of α-ANP in aqueous solution (pH = 7.4) collected at different times: from the bottom to the top t = 0 h, t = 5 h, t = 30 h, t = 168 h, t = 336 h.
(PPT)

Figure S10 **Representative infrared spectra of α-ANP in aqueous solution at pH = 4.0.** Representative infrared spectra of α-ANP in aqueous solution (pH = 4.0) collected at different times: from the bottom to the top t = 0 h, t = 1 h, t = 10 h, t = 48 h, t = 168 h.
(PPT)

Figure S11 **CR and anti-ANP immunostaining of heart specimens.** Images revealed the co-occurrence of CHF and IAA. CR-stained paraffin sections from left atrial appendages of three representative CHF patients showing atrial amyloid under direct light (**A, D, G**) and polarized light (**B, E, H**). **C, F, I:** Immunostaining of ANP in the atrial myocardium. Original magnification x 20.
(PPT)

Text S1 **Supporting Information.** This section presents detailed supporting materials to the manuscript and itemized, extensive results pertaining to the Infrared Analysis of ANP aggregation. Moreover, additional Figures as enhancement to those presented in the main text are shown.
(DOC)
