# Supplementary figures and images for: Different Factors Affecting Human ANP Amyloid Aggregation and Their Implications in Congestive Heart Failure
Source: PLoS One. 2011 Jul 26;6(7):e21870. doi: 10.1371/journal.pone.0021870 (PMC3144199; doi:10.1371/journal.pone.0021870)

## Slide 1
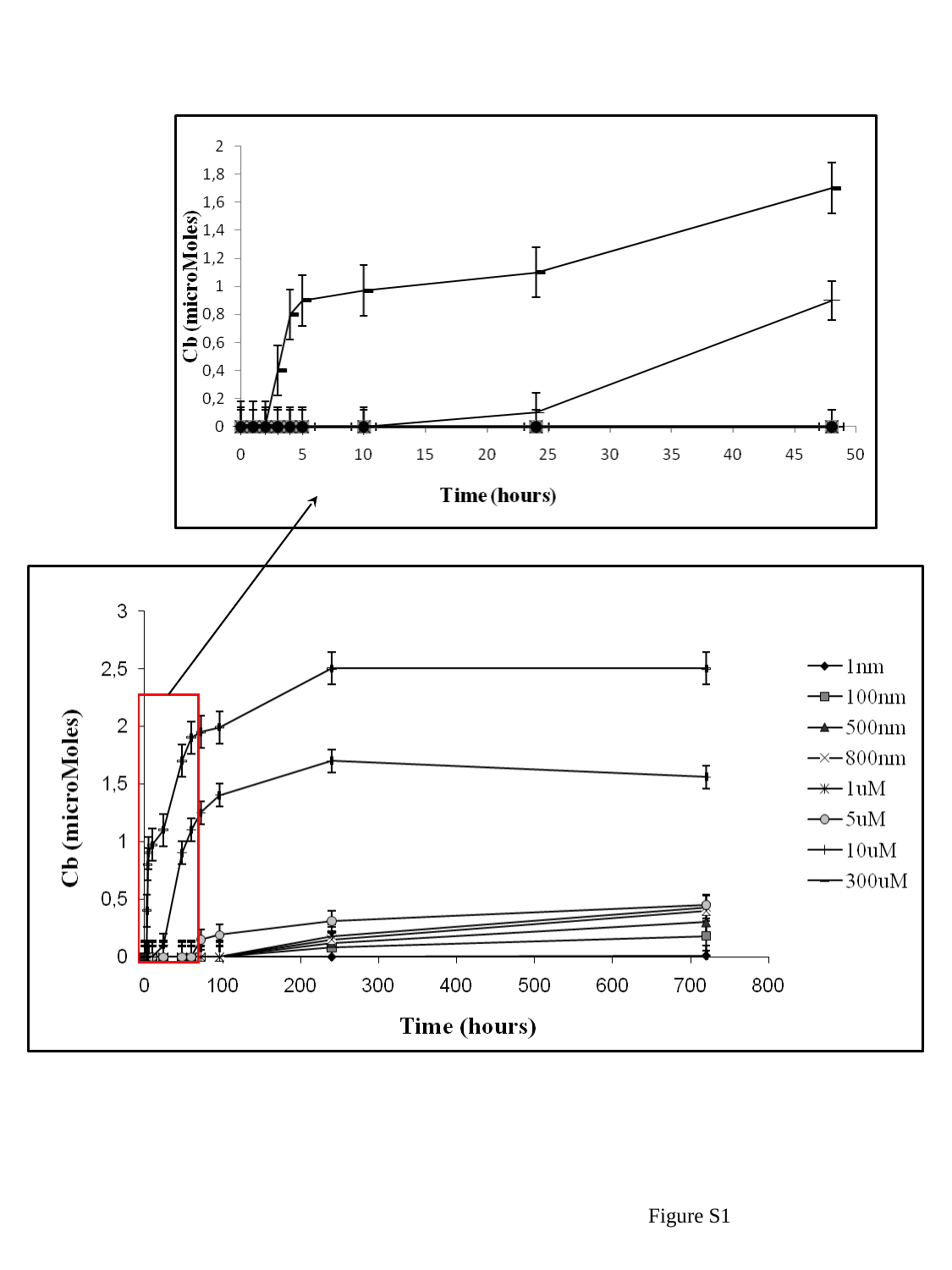

Figure S1

Supplement: Figure S1 — Aggregation kinetics via Congo Red. Time course of α-ANP aggregate formation using α-ANP previously disaggregated by treatment with HFIP (see Methods) and incubated at different concentrations at pH 7.4. Aggregate formation was measured by Congo Red binding method. Arrow indicate the magnification of red box area. (PPT) [file pone.0021870.s001.ppt]

## Slide 1
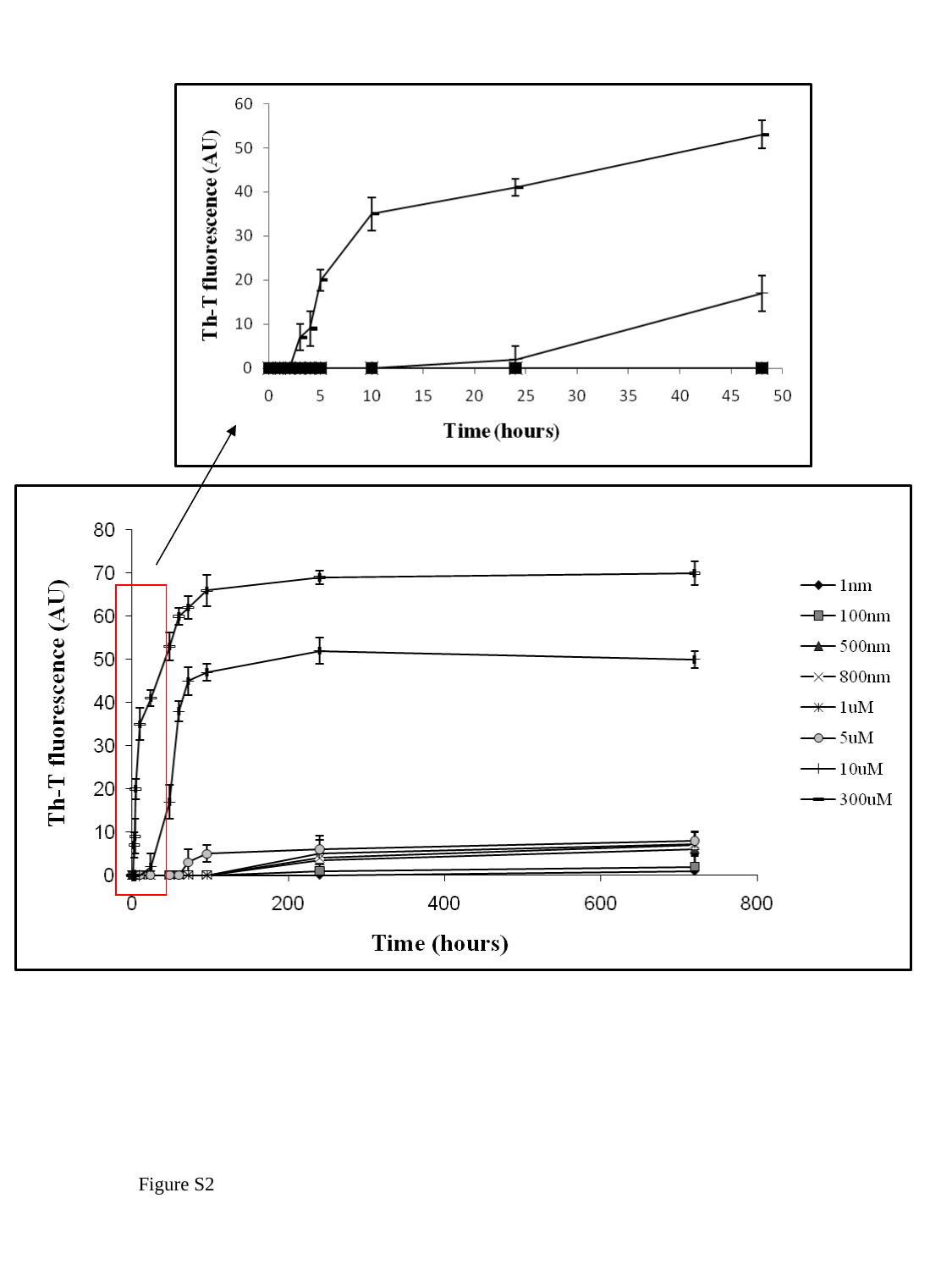

Figure S2

Supplement: Figure S2 — Aggregation kinetics via Th-T. α-ANP amyloid aggregation monitored by Th-T fluorescence. α-ANP HFIP treated was incubated at different concentration at pH 7.4. Arrow indicate the magnification of red box area. (PPT) [file pone.0021870.s002.ppt]

## Slide 1
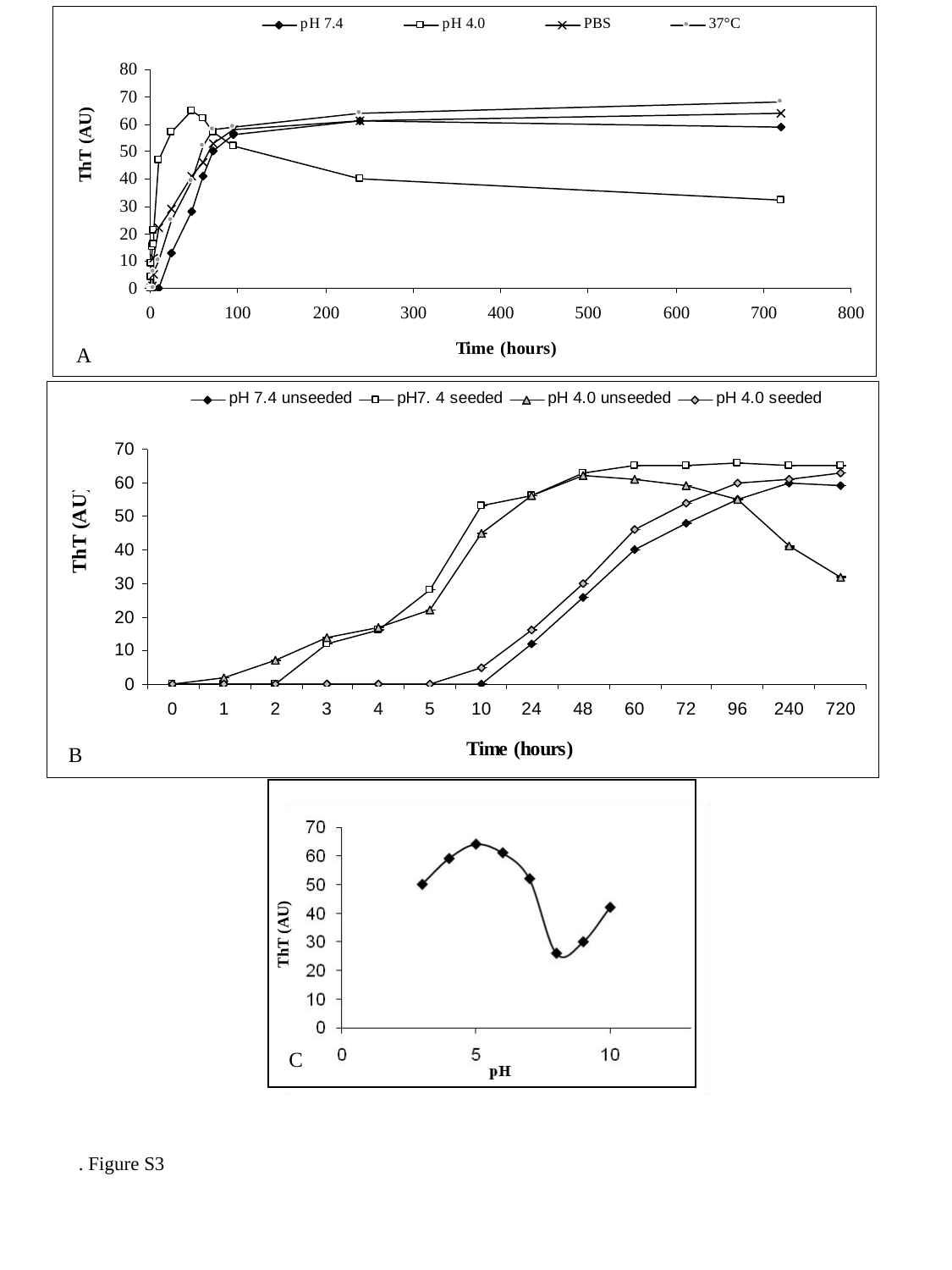

A
B
ThT (AU)
C
. Figure S3

Supplement: Figure S3 — Aggregation kinetics of α-ANP in different conditions. A) Effect of pH, solvent type and temperature on the aggregation kinetics of α-ANP measured via Th-T fluorescence. B) Seed-induced fibril formation of ANP in H2O (pH 7.4) at 25°C. The seeds or pre-existing fibrils were generated from aged ANP (pH 7.4 and pH 4.0 respectively) and were added as percentage weight fractions. Fibril formation was monitored over time by Th-T fluorescence. C) pH dependence of α-ANP aggregation. α-ANP at 0.5 mg/ml was incubated at 25°C at various pH values and after two days the amount of aggregation quantified by Th-T fluorescence. (PPT) [file pone.0021870.s003.ppt]

## Slide 1
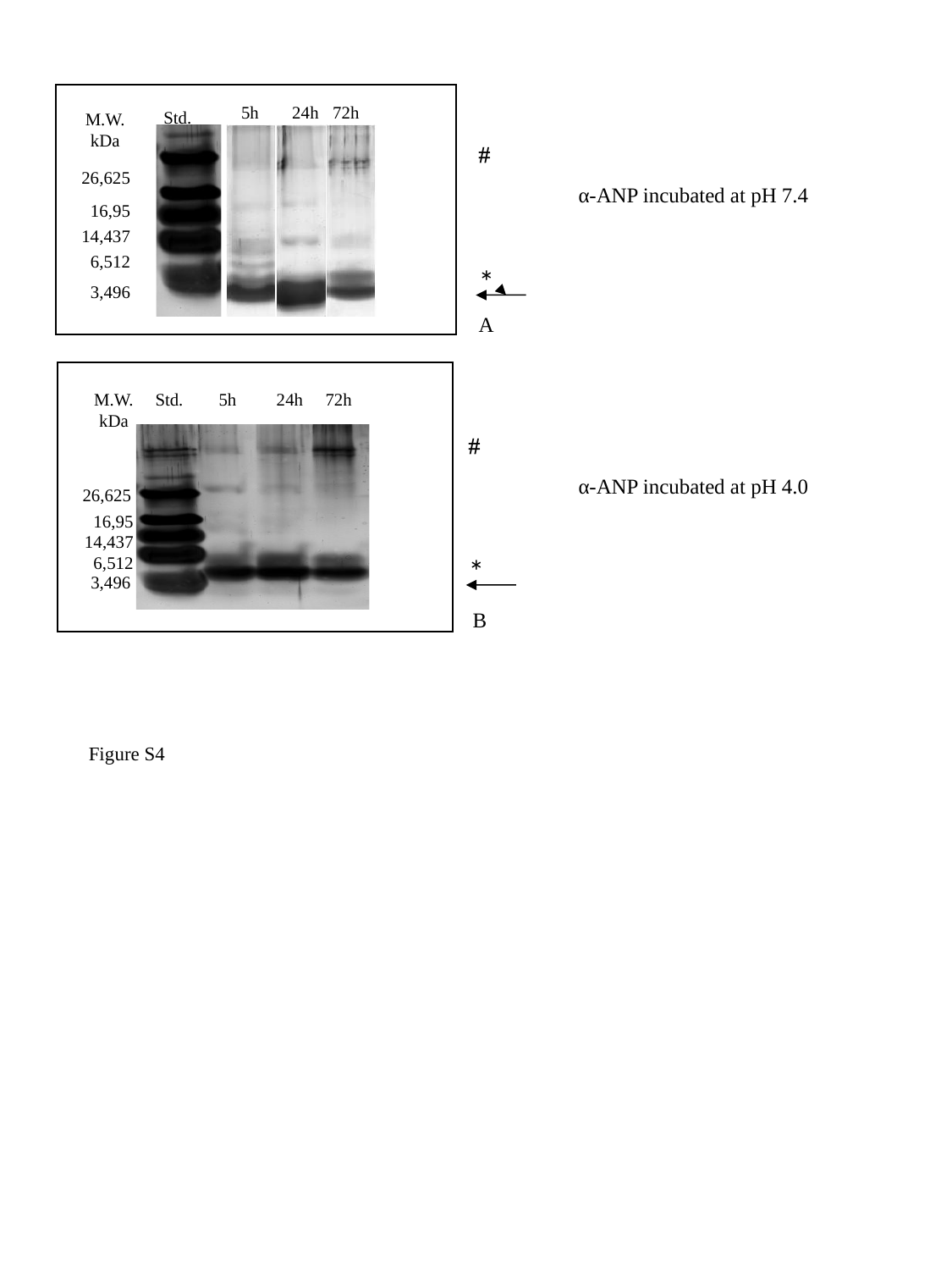

5h
24h
72h
Std.
M.W.
kDa

26,625
16,95
14,437
6,512
3,496
*
A
M.W.
kDa
Std. 5h 24h 72h

26,625
16,95
14,437
6,512
*
3,496
B
α-ANP incubated at pH 7.4
α-ANP incubated at pH 4.0
Figure S4

Supplement: Figure S4 — Formation of α-ANP oligomers and aggregates monitored by Tris-tricine SDS-PAGE. A) α-ANP solution in H2O at pH 7.4 at different aggregation times; B) α-ANP solution in H2O at pH 4.0 at different aggregation times; an appreciable difference in oligomers size and distribution between the two tested solutions was evident. Arrow indicates monomer, asterisk indicates dimer and # indicates high molecular aggregates. (PPT) [file pone.0021870.s004.ppt]

## Slide 1
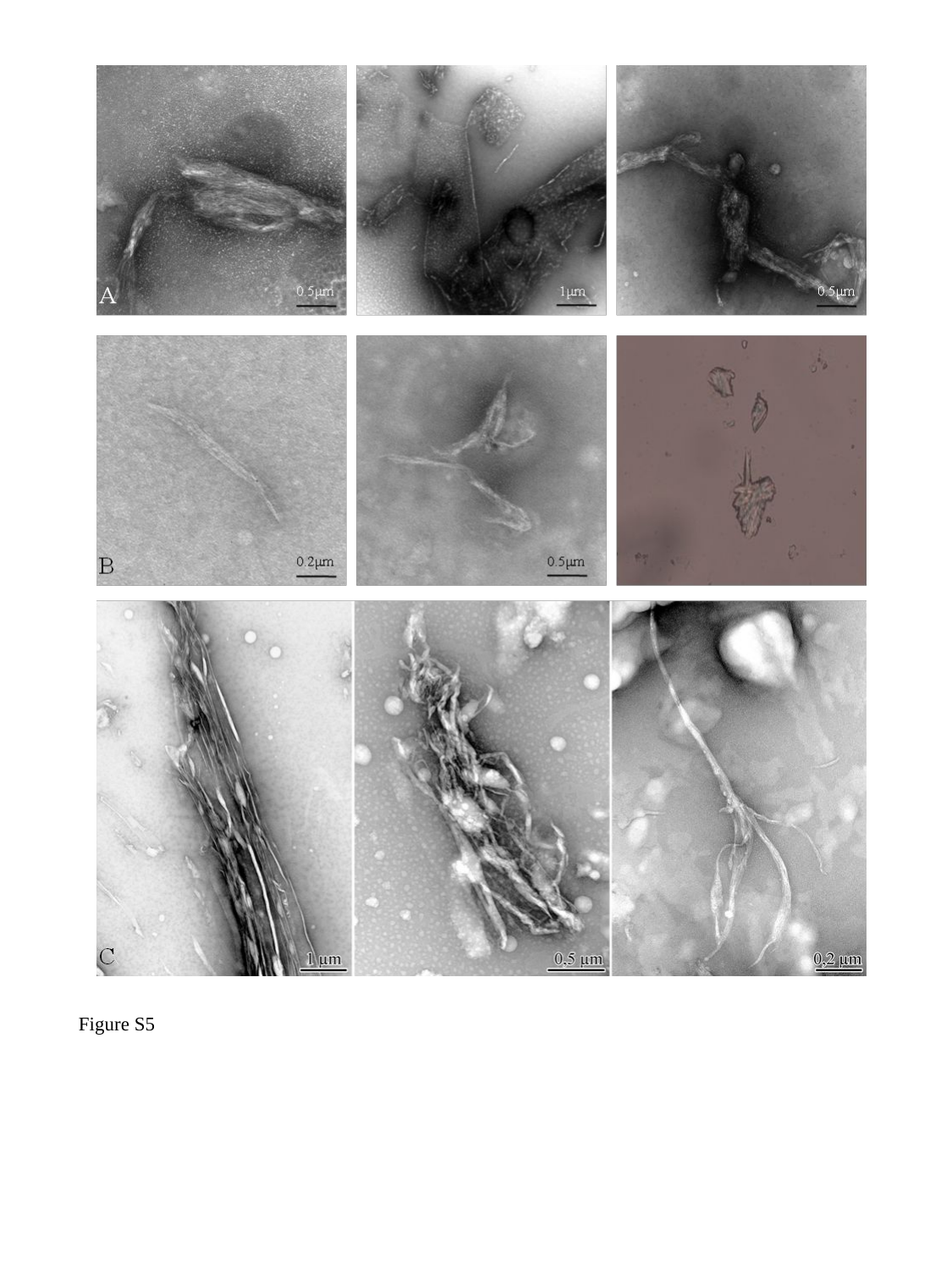

Figure S5

Supplement: Figure S5 — Microscope observations. A) CHF-ANP incubated at pH 7.4; Images show branched fibrils indicating that they are composed of interwined protofibrils and aggregates composed of fibrils with apparent different morphology. B) TEM micrographs of α-ANP incubated at pH 7.4: images show several fibrils that lie almost parallel and close to one another. One or more protofilaments unwinding from a fibril and winding onto an adjacent one are also observed. On the right, light microscopy of the α-ANP assembly is shown; CR brightfield (Magnification 20X); C) TEM observations of α-ANP incubated at pH 4.0 showing loose aggregates in which individual fibrils can be seen. Bundles of fibrils diverging from spherulites are evident. (PPT) [file pone.0021870.s005.ppt]

## Slide 1
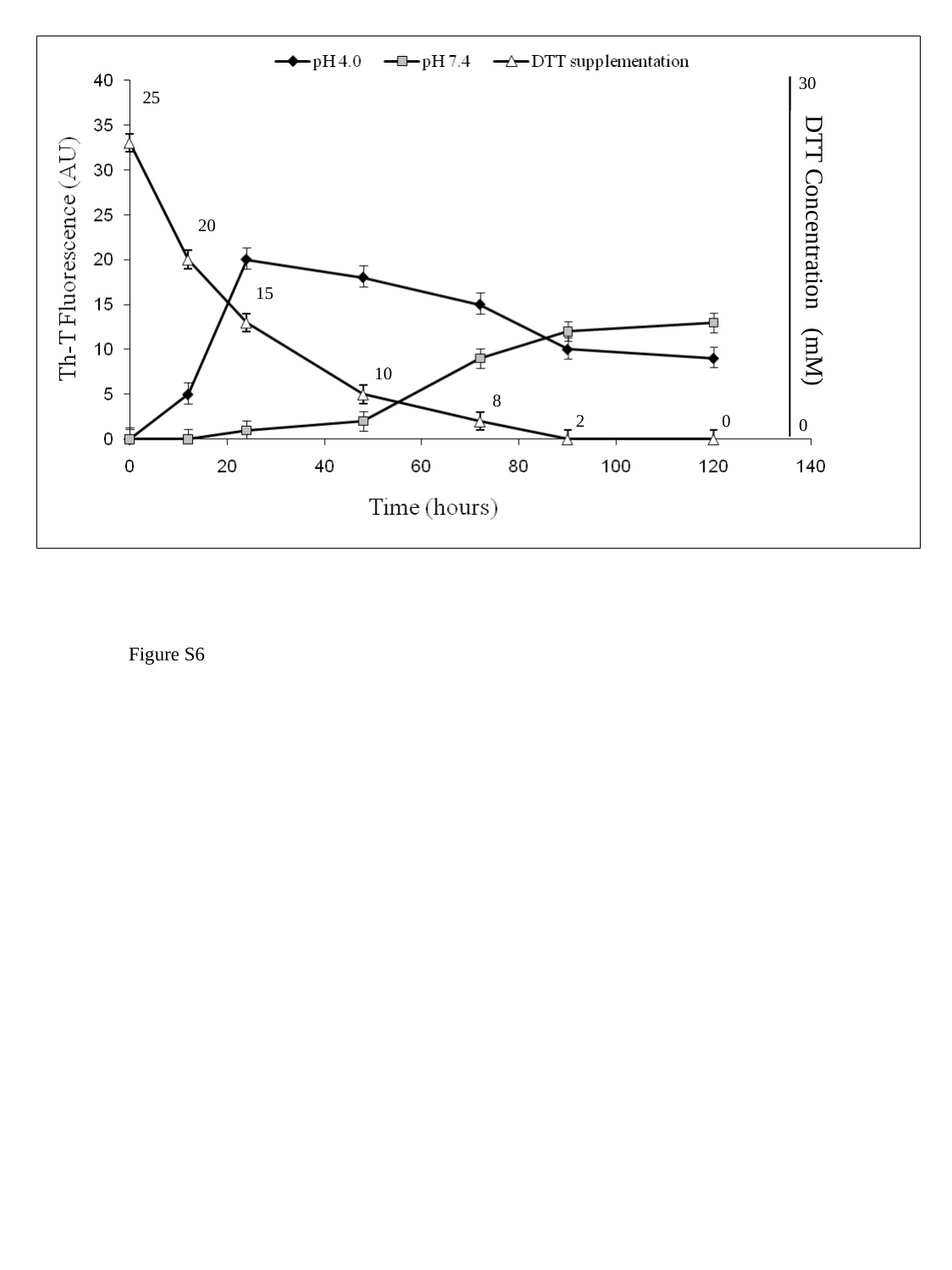

30
25
20
DTT Concentration (mM)
15
10
8
2
0
0
Figure S6

Supplement: Figure S6 — Effect of DTT addition on α-ANP incubated at pH 7.4 and pH 4.0. DTT is able to dose-dependently reduce the amount of amyloid fibrils formed under both pH 7.4 and pH 4.0 conditions. (PPT) [file pone.0021870.s006.ppt]

## Slide 1
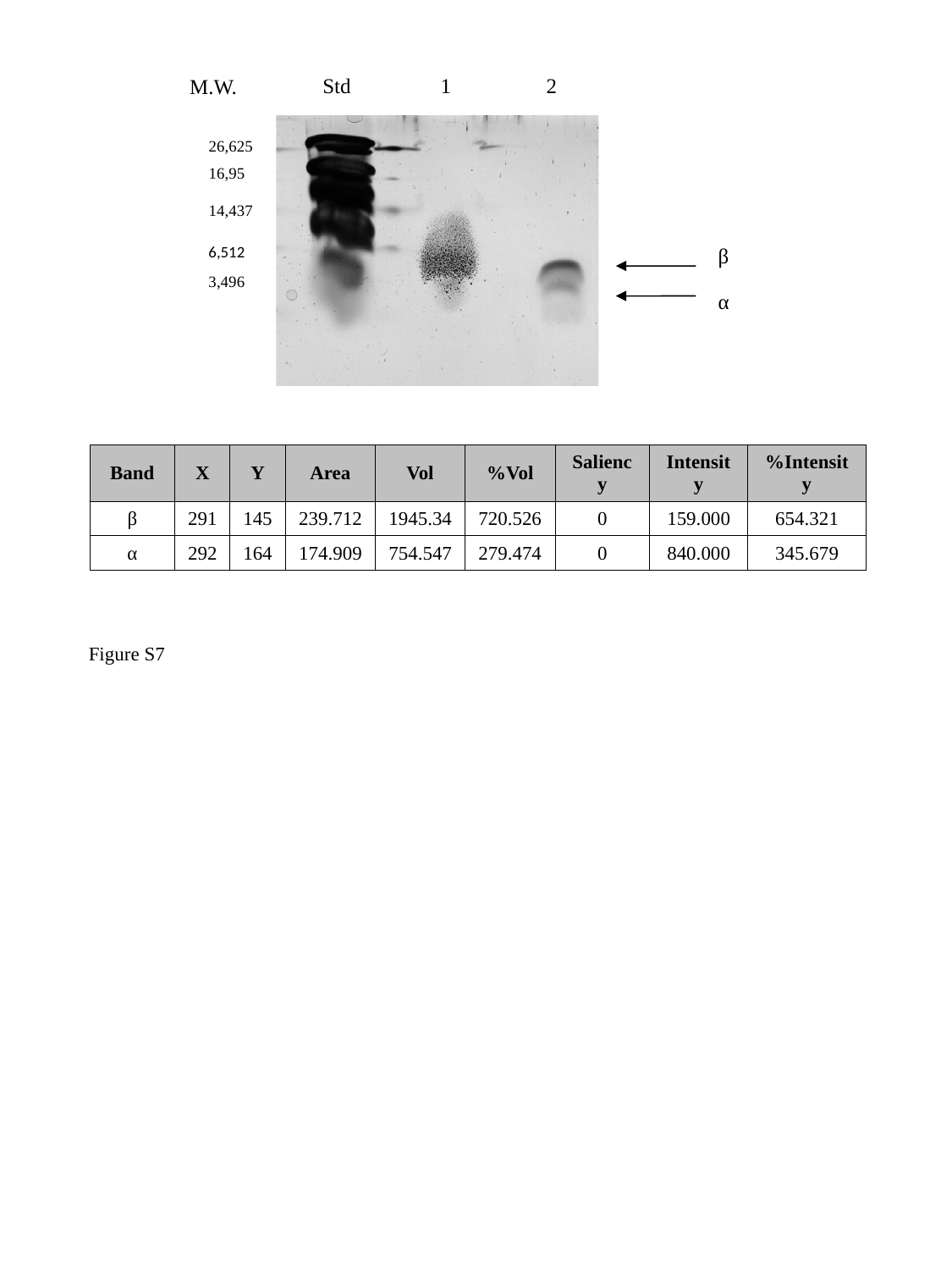

Std 1 2
M.W.
26,625
16,95
14,437
β
3,496
α
6,512
| Band | X | Y | Area | Vol | %Vol | Saliency | Intensity | %Intensity |
| --- | --- | --- | --- | --- | --- | --- | --- | --- |
| β | 291 | 145 | 239.712 | 1945.34 | 720.526 | 0 | 159.000 | 654.321 |
| α | 292 | 164 | 174.909 | 754.547 | 279.474 | 0 | 840.000 | 345.679 |
Figure S7

Supplement: Figure S7 — Electrophoresis. To model the conditions occurring in vivo in the heart of CHF patients, a mixture of ANP dimers and monomers (ratio 2∶1) roughly as in CHF conditions, was settled up. Figure shows Tricine SDS PAGE of ANP in H2O pH 7.4. 1) Untreated sample; 2) Treated sample. Quali-quantitative image analysis was performed on silver stained gel by using Image Quant (Master) software to assess the obtaining of monomer/dimer 1α-ANP:2β-ANP ratio as shown in the Table. (PPT) [file pone.0021870.s007.ppt]

## Slide 1
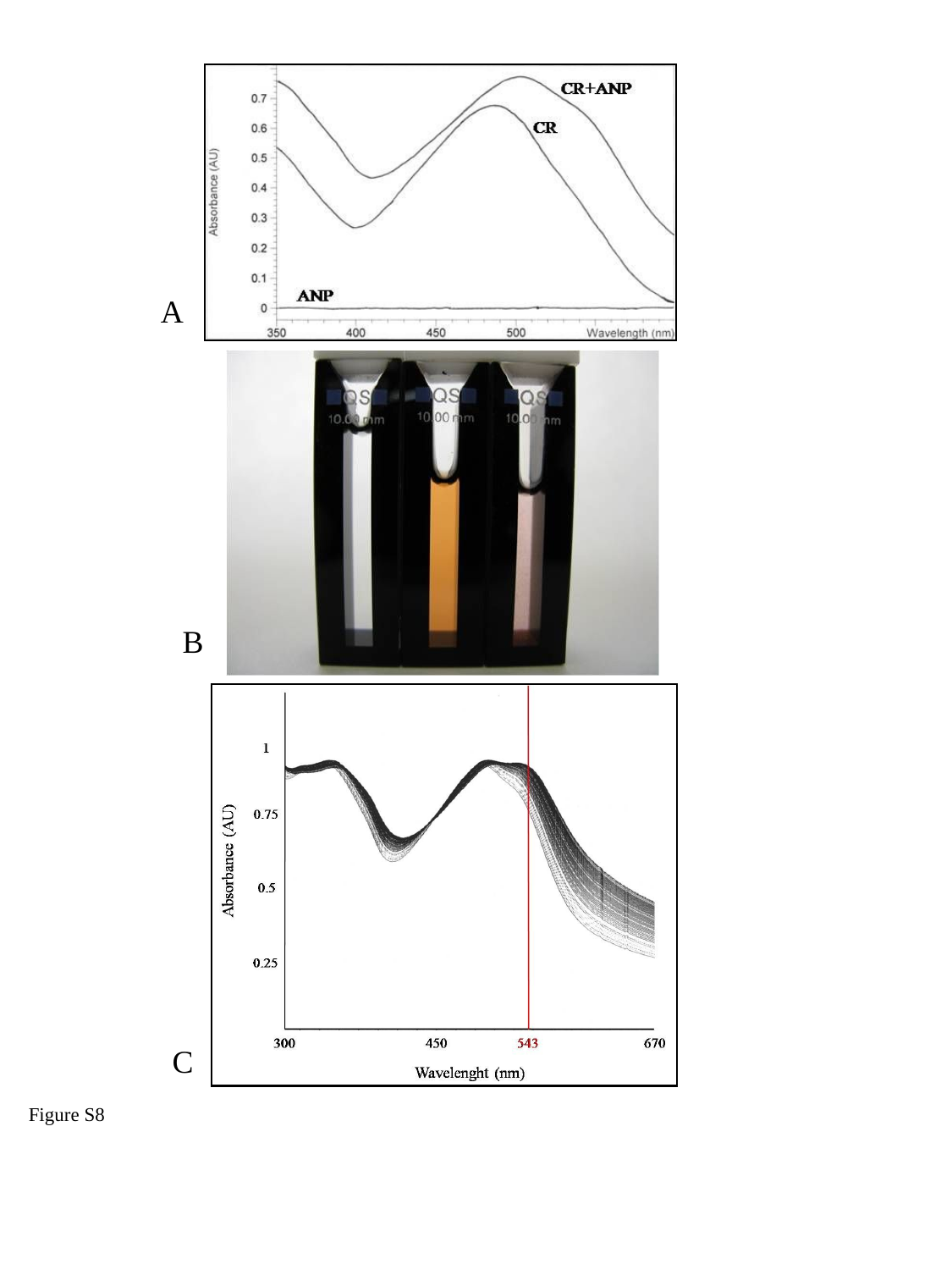

A
B
C
Figure S8

Supplement: Figure S8 — CR assay. A) Spectral features of CR and aggregated α-ANP. Absorbance spectra of a suspension of α-ANP in the absence and the presence of CR and of CR alone; B) When CR binds to excess fibrillar α-ANP, a change in colour from orange–red to rose is induced that corresponds to a shift in the characteristic absorbance spectrum of CR. C) Relationship between the shoulder peak (543 nm) and time, for 10 µM α-ANP in 20 µM CR. The original absorbance spectra at 541 nm at interval of 30 seconds on the aggregation of α-ANP are shown. The shoulder peaks at 541 nm gradually grew with time, indicating that CR is kinetically bound to the amyloid aggregates. (PPT) [file pone.0021870.s008.ppt]

## Slide 1
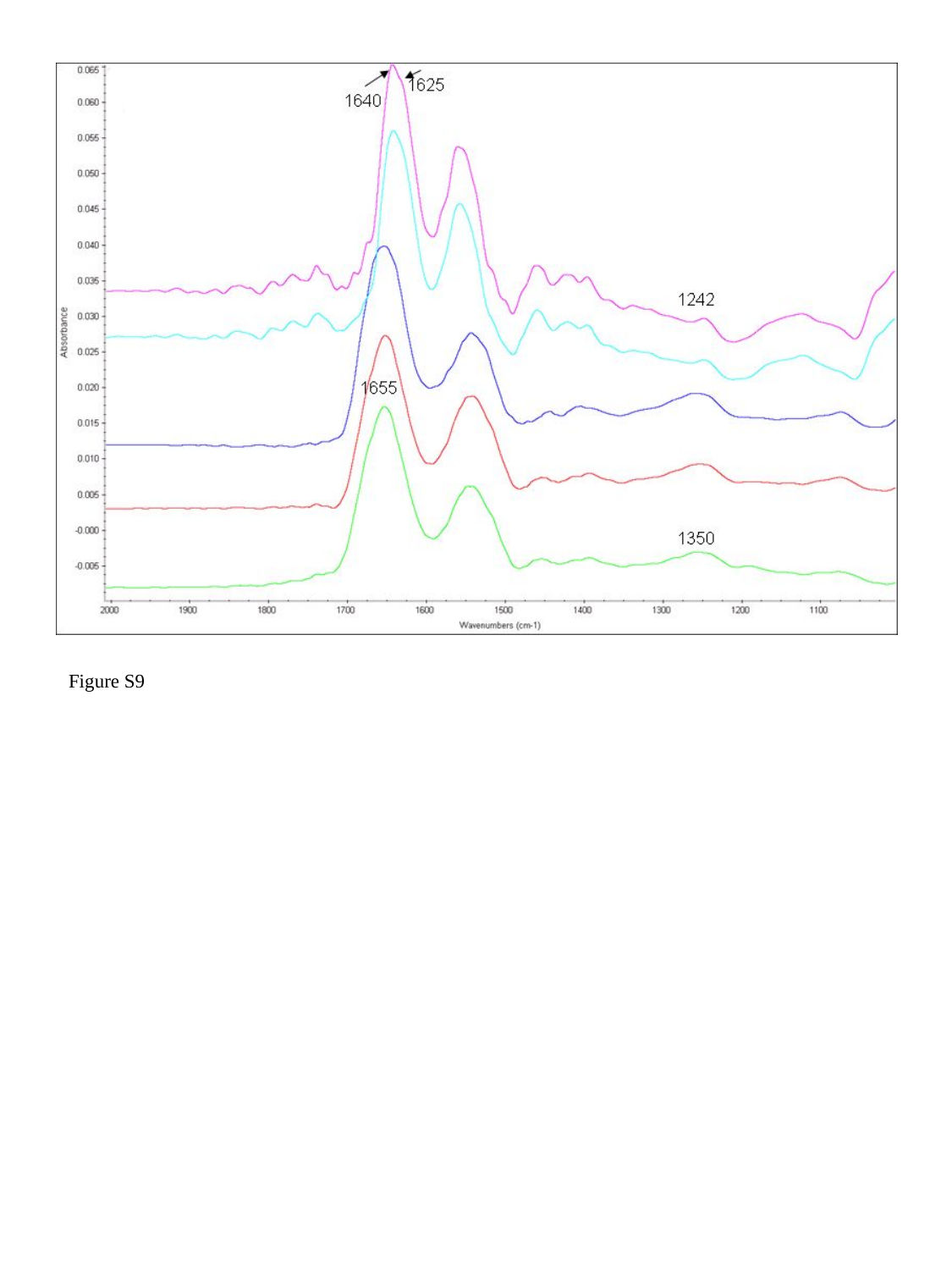

Figure S9

Supplement: Figure S9 — Representative infrared spectra of α-ANP in aqueous solution at pH = 7.4. Representative infrared spectra of α-ANP in aqueous solution (pH = 7.4) collected at different times: from the bottom to the top t = 0 h, t = 5 h, t = 30 h, t = 168 h, t = 336 h. (PPT) [file pone.0021870.s009.ppt]

## Slide 1
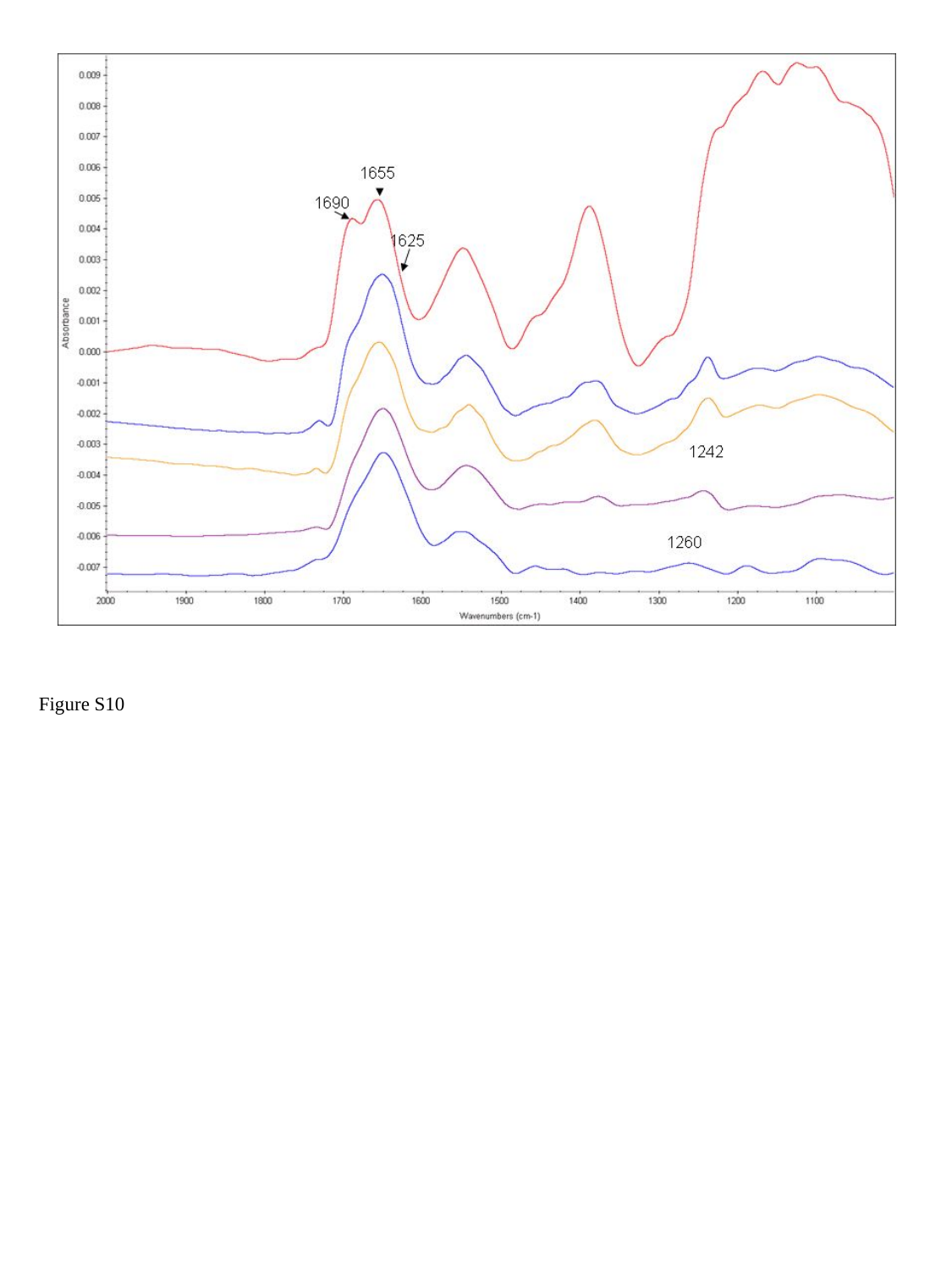

Figure S10

Supplement: Figure S10 — Representative infrared spectra of α-ANP in aqueous solution at pH = 4.0. Representative infrared spectra of α-ANP in aqueous solution (pH = 4.0) collected at different times: from the bottom to the top t = 0 h, t = 1 h, t = 10 h, t = 48 h, t = 168 h. (PPT) [file pone.0021870.s010.ppt]

## Slide 1
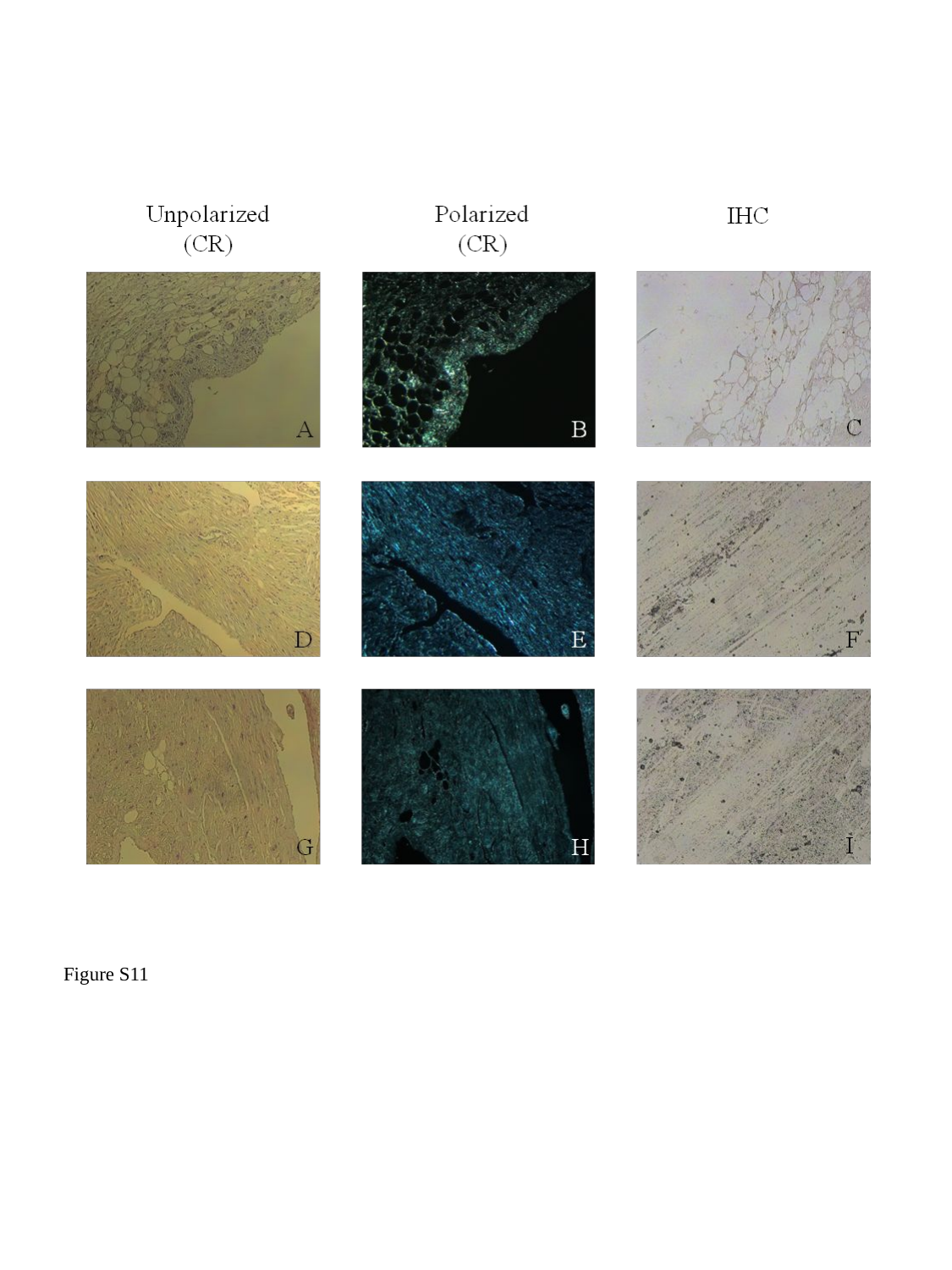

Figure S11

Supplement: Figure S11 — CR and anti-ANP immunostaining of heart specimens. Images revealed the co-occurrence of CHF and IAA. CR-stained paraffin sections from left atrial appendages of three representative CHF patients showing atrial amyloid under direct light (A, D, G) and polarized light (B, E, H). C, F, I: Immunostaining of ANP in the atrial myocardium. Original magnification x 20. (PPT) [file pone.0021870.s011.ppt]
