# Supplementary material for: Different Factors Affecting Human ANP Amyloid Aggregation and Their Implications in Congestive Heart Failure
Source: PLoS One. 2011 Jul 26;6(7):e21870. doi: 10.1371/journal.pone.0021870 (PMC3144199; doi:10.1371/journal.pone.0021870)
Supplement: Text S1 — Supporting Information. This section presents detailed supporting materials to the manuscript and itemized, extensive results pertaining to the Infrared Analysis of ANP aggregation. Moreover, additional Figures as enhancement to those presented in the main text are shown. (DOC) [file pone.0021870.s012.doc]

**SUPPORTING INFORMATION S1**

**Different Factors Affecting Human ANP Amyloid Aggregation and their Implications in Congestive Heart Failure**

Lia Millucci^1^ PhD, Eugenio Paccagnini^2^ PhD, Lorenzo Ghezzi^1^, Giulia Bernardini^1^ PhD, Daniela Braconi^1^ PhD, Marcella Laschi^1^, Marco Consumi^3^ PhD, Adriano Spreafico^4^ PhD, Piero Tanganelli^5^ MD, Pietro Lupetti^2^ PhD, Agnese Magnani^3^ PhD and Annalisa Santucci^1^ PhD ^*^

*^1^ Dipartimento di Biotecnologie, Università degli Studi di Siena, Siena, Italy; ^2^ Dipartimento di Biologia Evolutiva, Università degli Studi di Siena, Siena, Italy; ^3^ Dipartimento Farmaco Chimico Tecnologico, Università degli Studi di Siena, Siena, Italy; ^4^ Dipartimento di Medicina Clinica e Scienze Immunologiche, Università degli Studi di Siena, Siena, Italy; ^5^ Dipartimento di Patologia Umana e Oncologia, Università degli Studi di Siena, Siena, Italy*

***Address for correspondence: Prof. Annalisa Santucci

Dipartimento di Biotecnologie

Università degli Studi di Siena

via Fiorentina 1, 53100, Siena, Italy

Tel: 00390577234958

Fax: 00390577234903

e-mail: [santucci@unisi.it](mailto:santucci@unisi.it)

**Detailed Methods**

**Congo Red amyloid detection and anti-ANP immunoreactivity in CHF heart specimens**

Left atrial appendages were obtained from 40 CHF patients (**Table 1**) undergoing cardiac transplantation, older than 18 years of age and scheduled for heart transplantation. The investigation conformed with the principles outlined in the Declaration of Helsinki and was conducted following the approval of the local University Hospital Ethics Committee (“Comitato Etico Locale dell’Azienda Ospedaliera Universitaria Senese”).

Age, sex, and the presence or absence of hypertension, diabetes mellitus, and atrial fibrillation were recorded from case notes (**Table 1**).

Four standard tissue blocks were taken from the each patient atrium. After fixation in buffered formalin, 3μm sections were stained by Congo Red (CR) and the type of amyloid detected was confirmed by its immunoreactivity with human atrial natriuretic peptide (ANP) antibody (dilution 1:200 and
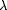
-light chain (1:200), from Santa Cruz Biotechnology, Santa Cruz, CA. The degree of IAA deposition in the atrial wall was graded subjectively for each heart (by an examiner who was blinded to the clinical and autopsy data of the deceased) on a scale of 0–3 according to the following criteria: grade 0=not present; grade 1=occasional small specks and fine fibres around cardiomyocytes; grade 2=a moderate number of deposits through the entire thickness of the myocardium; grade 3=a dense network of fibers. For each heart, the highest grade from the sites evaluated was taken as representative (Figure S11).

**Peptide.** Synthetic ANP was purchased from AnaSpec Inc.

**α-ANP preparation.** Lyophilized peptide was stored in sealed glass vials in desiccated containers at -80 °C. Prior to resuspension, each vial was allowed to equilibrate at room temperature for 30 min to avoid condensation upon opening the vial. The first step in resuspending the lyophilized peptide was treatment in 1,1,1,3,3,3-hexafluoro-2-propanol (HFIP; catalog number H8508; Sigma). All work with HFIP was done in a chemical fume hood with adequate protection. 1 mg of peptide was diluted in 100% HFIP to 1 mM using a glass gas-tight Hamilton syringe with a Teflon plunger. The clear solution containing the dissolved peptide was then aliquoted in microcentrifuge tubes (VWR, 20170–293) using a positive displacement repeat pipette (Multipette, Eppendorf). The HFIP was allowed to evaporate in the fume hood, and the resulting clear peptide films were dried under vacuum (6.7 mtorr) in a SpeedVac (Savant Instruments) and stored desiccated at -80°C. To confirm the complete ANP dissociation in to monomers after this treatment, reverse phase HPLC was carried out with 0.1% TFA as the mobile phase. The HPLC chromatogram showed a sharp peak with an apparent molecular mass of approximately 3 kDa. No significant change was detected when the sample was injected one day after preparation (data not shown). α-ANP in HFIP was completely dissociated into monomers. Under our experimental conditions, HFIP was the only solvents able to yield monomeric α-ANP solutions. Immediately prior to use, the HFIP-treated aliquots were carefully and completely resuspended to the desired concentration in the appropriate buffer.

Concentrations of ANP were determined by UV absorbance with a computed extinction coefficient of 1490 cm^−1^ M^−1^ at 280 nm [S1]

All reactions and measurements were carried out at room temperature (25 °C) except for otherwise specified.

Aggregation experiments were performed at α-ANP concentrations in the 1nm-300μM range; in the case of aggregation at acidic pH, we performed experiments after acidification of the peptide solution at pH 4.0. To ensure that the addition of the small amounts of dilute base or acid did not alter the peptide concentration, quantitative amino acid analysis was performed at the beginning and end of the study for all samples, which established that the peptide concentrations remained essentially unchanged (±5%).

**α-ANP aggregation with SDS**

0.5mM SDS solutions (this concentration of SDS is very close to the critical micelle concentration (CMC) of SDS in physiologic saline at 28°C [S[2](http://jdr.sagepub.com/content/86/7/618.full#ref-9%23ref-9)]) were filtered (0.2 μm cellulose acetate filters, VWR Scientific Products, West Chester, PA) and were sonicated (30 s) in a bath sonicator 20 min prior to being mixed with α-ANP. Reactions were initiated in siliconized Eppendorf tubes by incubating appropriate concentrations of freshly prepared α-ANP monomer in 10 mM PBS in the presence of 0.5mM SDS without agitation. Aggregation kinetic parameters were obtained by monitoring the reaction with Congo Red (CR) method.

**ANP-CHF preparation-** In CHF a plasma monomer/dimer ratio of 1α-ANP:2β-ANP (ANP-CHF) is found [S3]. This in solution conditions were reproduced following our original protocol, as follows. Lyophilized ANP was dissolved in H_2_O pH 7.4 at a concentration of 1mg/mL and centrifuged at 16,500 x *g* for 1h to remove large aggregates. The supernatant was collected and filtered with a 20nm pore filter; protein concentration was then determined at 280 nm using an Agilent 8354 spectrophotometer [S1].

Tricine SDS-PAGE was adopted to assess the obtaining of monomer/dimer 1α-ANP:2β-ANP ratio immediately after filtering supernatant . Quali-quantitative image analysis was performed on silver stained gel according to [S4] by using Image Quant (Master) software (Figure S7). Aliquots of ANP-CHF were snap-frozen in liquid nitrogen and stored at −80 C.

**Electrophoresis**

***Glutaraldehyde cross-linking*** - Glutaraldehyde reacts with the amino group of lysine side chains and therefore has been used extensively to cross-link oligomeric proteins [S5, S6]. The glutaraldehyde cross-linking reaction was adopted to block α-ANP oligomeric forms at the initial aggregation times, according to [S7], with some modifications. All cross-linking reactions were conducted in H_2_O at pH 7.4 or 4.0 depending on the analyzed sample and uncontrolled room temperature of approximately 295 K. α-ANP samples (10μM) in H_2_O were reacted with 0.06% (w/v) glutaraldehyde in capped centrifuge tubes. The reactions were stopped after 20min by adding a tenfold molar excess of hydrazine to glutaraldehyde which depleted any unreacted glutaraldehyde. The cross-linked α-ANP samples were analyzed by SDS-PAGE. Final products were revealed by Coomassie blue dye and silver staining in order to detect the protein bands with a higher sensitivity.

***Detection of aggregates -*** The conditions used to cross-link the α-ANP solutions were chosen while considering that high protein concentrations and high glutaraldehyde concentrations can result in non-specific inter-aggregate cross-linking and erroneously high aggregate concentrations. For this reason, the molar ratio of glutaraldehyde to protein concentration was kept constant and the total protein concentration was kept low.

***Tricine SDS-PAGE*** - Oligomerization of α-ANP (10μM) incubated in H_2_O at pH 7.4 or pH 4.0 for times longer than 1 hour, was examined by 4–20% gradient Tris/tricine SDS-PAGE according to [S8].

***SDS-PAGE. -***.The identification of occurring α-ANP multimers at 48 hours and 30 days incubation (final aggregation states of α-ANP incubated at pH 4.0 and pH 7.4 respectively) was achieved by SDS-polyacrylamide gel electrophoresis on Criterion Bis-Tris 12% with XT MES running buffer (BIO-RAD). Using this protocol, we consistently detect α-ANP monomers, dimers, trimers, tetramers, and higher molecular weight multimers.

**α-ANP and ANP-CHF Fibril Formation.** α-ANP or ANP-CHF samples were dissolved at different concentrations in H_2_O adjusted at pH 4.0 or in PBS 10mM pH 7.4. Incubations were carried on for 30 days at 25°C or 37°C. Aggregation kinetic parameters were obtained by monitoring the reaction with Congo Red (CR) and Thioflavin T (Th-T).

**Congo Red Assay.** The time course of α-ANP aggregation was conveniently monitored with CR. In general, the procedure described by Klunk et al.[S9] was followed, but with minor modifications (Figure S8). A Congo Red stock solution (20 mM) was prepared in PBS/ ethanol (9:1, v/v) at pH 7.4 and filtered to remove micelles. From this stock solution, a second, diluted Congo red solution was prepared (20 μM), for which an accurate concentration was determined by absorbance spectroscopy at 505 nm and a standard Beer–Lambert plot (5.53 10^4^ (absorbance unit) cm^–1^ M^–1^). Aliquots (25 μl) taken from each tested α-ANP solution of appropriate concentration at the desired pH were combined with 1.0 ml of the Congo Red solution (20 μM), and incubated at room temperature for 15 minutes. Congo red binding was ascertained by measurement at 300–700 nm using a Agilent 84353 spectrophotometer, with subtraction of the baseline spectrum of the phosphate buffer. The absorbance spectrum of bound Congo red plus peptide was corrected for the light-scattering by subtracting the absorbance spectra of ANP taken over the visible region. The final spectra were then compared to that of non-bonded Congo red solution. The amount of CR bound (Cb) was calculated as follows:

*Cb*[M] = (*A*_540_/25,295) - (*A*_480_/46,306)

Three independent experiments were performed for each sample.

**Th-T assay.** Th-T assay was performed according to [S10]. The Th-T fluorescence measurements were performed on a F-4500 Fluorescence spectrophotometer, using excitation and emission slit-widths of 5 nm. For the Th-T binding analysis, fluorescence intensity was measured at 450 nm excitation and 480 nm emission wavelengths. Sealed 1 cm × 1 cm × 4.5 cm polystyrene cuvettes were utilized. At specific time-points, aliquots (20 μl) of ANP solutions at the desired concentration and pH values, were mixed thoroughly with a Th-T solution (0.48 ml, 10 μM) containing potassium phosphate buffer pH 6.0, with immediate measurement of the fluorescence. The signal was normalised by dividing the observed signal by that of the buffer alone containing Th-T. Control experiments showed that the inclusion of Th-T in the buffer does not alter the fibril growth kinetics or the morphologies of the fibrils formed.

**FTIR measurements**

The spectra were obtained with a Thermo Nicolet 5700 Fourier Transform Infrared spectrometer, operating between 3000 and 900 cm^-1^. An MCT detector was used and the apparatus was purged with dry nitrogen. Typically, 100 scans at a resolution of 2.0 cm^-1^ were averaged. The frequency scale was internally calibrated with a reference He-Ne laser to an accuracy of 0.01 cm^-1^. An ATR (Attenuated total reflectance) cell for liquid equipped with a 45° germanium IRE (internal reflection element) crystal was used to record the spectra of water and ANP aqueous solution.

The α-ANP solutions for the IR experiments was obtained by dissolving 0,5 mg of α-ANP in 1.0 mL of deionised water adjusted to the desired pH just before to start the acquisition of IR spectra.

*Spectral Processing*

The spectra were taken in a single beam mode at predetermined time intervals to obtain kinetic information of the peptide aggregation process. The spectra of both water and α-ANP solution were collected first. Then the spectra of α-ANP were obtained by subtracting the spectrum of water from that of the sample solution and correcting the difference spectrum for the peptide adsorbed at the surface of the ATR crystal. The scale factor for subtraction of water spectrum was chosen so that the spectral region between 2000 and 1700 cm^-1^ was flat.

*Spectral Enhancement*

In order to improve the observability of the overlapping bands, mathematical resolution enhancement was performed by a spectral deconvolution process that is similar to the Fourier self-deconvolution, except that the mathematical operations are performed in the spectral domain rather in the Fourier domain. The spectral deconvolution process moves intensity from the outer wings of a band to the center of the band, therefore reducing its effective half-width and improving its observability. The quality of the deconvolution procedure is controlled by two variables, namely the “half-band width” of the Lorentzian line used for deconvolution and the “resolution enhancement” achieved.

**Purification of α-ANP fibrils.** For analysis by light microscopy and fibril seeding, fibril samples were washed with deionized water 10 times to remove any buffer’s components, as we determined that in some case they were tightly bound to the fibrillar material. Washing steps included centrifugation of liquid fibril suspensions at 14.100 x g for 10 min, discarding the supernatant, followed by resuspension in deionized water, and shaking for 10 min. Samples were then air-dried to remove water, resulting in a flaky-white material.

**Cross-Seeding Experiments**

Sonicated fibrils of α-ANP incubated at ph 4.0 and pH 7.4 were used as seeds in cross-seeding experiments. Seeds were prepared according to [S11] with minor modifications: a solution of fibrils (1 mg/mL) was centrifuged at 14

000 rpm for 10 min. After the supernatant was removed, the pellet was suspended in water, shaking for 10 min and mildly sonicated. The sonicated seed fibrils were short, 100−300 nm, on the basis of electron microscopy (EM) images (data not shown). The effect of cross-seeding on the kinetics of fibrillation was studied by adding 4% (w/w) of the seeds. Finally, 20 μM CR was added to each sample and samples were incubated at 25 °C. The same samples were analyzed by Th-T assay.

**DTT dose-dependent reduction of α-ANP aggregation -** Increasing amounts of DTT (0–25 mM) were added to aliquots of 10μM pH 7.4 or pH 4.0 α-ANP and tested for Th-T fluorescence.

**Disaggregation assay of fibrils.** To test the resistance of fibrils formed under different conditions, aliquots of mature fibrils suspensions were centrifuged at 14.100 x g for 20 min at room temperature and resuspended in guanidine hydrochloride (GdnHCl ) concentrations ranging from 0 to 8M [S12]. After 1 h incubation, samples were centrifuged at 14.100 x g for 20 min at room temperature, and the fraction of protein present in the supernatant, which is a measure of how much protein is resolubilized from fully formed fibrils by GdnHCl, was determined [S13].

**Transmission Electron Microscopy.** For analysis by transmission electron microscopy (TEM), fibril samples were suspended in deionized water and then diluted 100-fold. 5 μL of each sample were placed on a glow-discharged 200 mesh carbon coated copper grid. After adsorption for 1 min, the samples were washed with distilled water and air-dried. The grids were then stained with 2 % uranyl acetate for 45 s. Excess stain was removed, and the samples were again allowed to air-dry. The samples were analyzed utilizing a Philips CM10 TEM operating at 80 kV.

**Supplementary Results**

**Infrared analysis**

Infrared spectroscopy was used to study the ANP aggregation in aqueous solution with the aim to gain both kinetic and structural information on the peptide aggregation process.

Infrared spectroscopy coupled with attenuated total reflectance technique is a powerful tool to study proteins and peptides conformation in aqueous solution and it can also provides useful information on the kinetic of several processes such as adsorption to surfaces, denaturation and aggregation.

Two different pH conditions (pH = 7.4 and pH = 4.0) were tested in order to evaluate the effect of physiological or more-denaturing conditions either on peptide secondary structure or aggregation rate.

The infrared spectrum of a peptide is characterised by specific regions relative to the vibrational modes of the peptide group (NH-CO): [Amide I (1700-1600 cm^-1^), Amide II (1600-1500 cm^-1^), Amide III (1380-1200 cm^-1^)]. The covalent bonds of the peptide group vibrate at different wavenumbers as a function of both peptide secondary structure and H-bonding concentration within the macromolecule. Amide I and III are very sensitive to the peptide secondary structure, thus the analysis of this spectral regions can be used to follow the α-ANP aggregation process in aqueous solution.

**Figure S9:** Representative infrared spectra of α-ANP in aqueous solution at pH = 7.4.

**Figure S10:** Representative infrared spectra of α-ANP in aqueous solution at pH = 4.0.

In our experiments we observed spectral changes in both Amide I and Amide III regions with time suggesting that the peptide underwent structural variations. In particular, the infrared spectrum of α-ANP in aqueous solution at pH=7.4, collected at t=0, exhibits an intense wide Amide I band at approximately 1655 cm^-1^ which drops to lower wavenumbers (1640 cm^-1^) after 168 hours. Moreover, in the Amide III region a drop of the 1350 cm^-1^ maximum to 1242 cm^-1^ was observed too.

Similar variations, even with a more rapid kinetics, were observed for α-ANP in aqueous solution at pH 4.0.

These findings strongly suggest that secondary structure changes occur for the peptide with time leading to the formation of β structures.

Amide I region was selected to follow the aggregation process of α-ANP, because of higher intensity of this band with respect to the Amide III one.

Fourier-self deconvolution was applied to the Amide I band in order to increase the observability of the single spectral contributions and follow their intensity variation with time. The representative Fourier-self deconvoluted Amide I spectral region of α-ANP in aqueous solution collected at different times under two different pH conditions (7.4 and 4.0) are shown in Fig. 2.

pH = 7.4

The deconvoluted spectra showed a slow decrease of the 1655 cm^-1^ intensity with time, accompanied by the intensity increase of the two shoulders at 1635 and 1675 cm^-1^. These two absorption bands were attributed to the packing of β strands into β-sheets and to the subsequent formation of intermolecular β-sheet aggregates. In our sample these two shoulders reached the maximum intensity (then remaining constant) after 168 hours (7 days), revealing that the formation of these intermolecular β-sheet aggregates occurs with a very slow kinetics. After this period of time the infrared spectrum of the peptide does not undergo to any further change indicating that the formed aggregates were stable, whereas the aqueous solution was converted into a gel-like system.

pH = 4.0

At pH 4.0 the 1625 and 1690 cm^-1^ contributions (attributed to β-sheet structures and intermolecular β-sheet aggregates) of the Amide I band immediately appear (they are detectable within the first acquired IR spectrum) in the spectrum of ANP in aqueous solution and their intensity increases quickly. The intensity at 1625 and 1690 cm^-1^ reaches a maximum after about 10 hours, then remaining constant up to 48 hours, indicating that the aggregation kinetics at this pH is significantly higher than at the physiological one. The lack of significant changes in the spectra registered between 10 and 48 hours suggests that the aggregated structures are quite stable within this period.

The ANP IR spectra recorded from 48 to 168 hours are instead not any more superimposable to each other indicating that the aggregated structures are not so stable under these conditions after 48 hours, according to the results obtained by microscopic observations. Moreover, the ANP solution after 168 hours still looks like a solution and does not resemble the gel-like system observed at pH 7.4. Unlike pH 7.4, in the deconvoluted spectra of the aggregate structures the 1690 cm^-1^ contribution becomes much more intense than that at 1625 cm^-1^ with time and the Amide I band more asymmetrically broaden, indicating a large increase of random coil structures with time.

Secondary structure of ANP is not known yet. The structure of the mutant ANP (124-151, 28 aa) has been resolved and it shows β-strands secondary structure at the 139-141 positions [S14]. Previous conformational studies of an ANP mutant demonstrated for the peptide a inherent flexibility in solution (this is confirmed by our infrared studies of ANP in D_2_O (data not shown) which demonstrated a very rapid H/D exchange for all the peptide protons suggesting that they are not involved in H-bonding interactions) and the involvement of the C-terminal tail in hydrophobic packing [S**14**].

Our infrared data confirm that ANP aggregation in aqueous solution occurs trough the packing of β strands into β-sheets followed by the formation of intermolecular β aggregated structures, (amyloid fibrils). The kinetics of the peptide aggregation process in aqueous solution as well as the stability of the β aggregated structures are influenced by the environmental conditions as solution pH. In fact, the infrared data show that the ANP aggregates form in a week at pH 7.4, whereas only 10 hours are needed for the ANP β strand packing at pH 4.0. At pH 7.4, the β aggregates, once formed, do not change their secondary structure with time (the infrared spectra collected after 168 hours are all superimposable), whereas at pH 4.0 after 48 hours secondary structure changes occur (infrared spectra change with time revealing an increase of random coil structures) suggesting that additional peptide-peptide interactions may contribute to the aggregation mechanism and influence the amyloid fibrils stability.

The infrared data suggests that ANP aggregation in aqueous solution does occur trough the packing of β strands into β-sheets followed by the formation of intermolecular β aggregated structures. The aggregation process occurs with a very slow kinetics under physiological conditions and the aggregated structures formed after a week do not change secondary structure with time. The kinetics of peptide aggregation is accelerated by lowering the solution pH, but the stability of the aggregated structures is compromised.

**Supplementary References**

1. ProtParam on ExPASy Proteomics Server (<http://expasy.org/tools/protparam.html>)
2. Necula M, Chirita CN, Kuret J (2003) Rapid anionic micelle-mediated alpha-synuclein fibrillization in vitro. J. Biol. Chem 278: 46674-46680.
3. Sugawara A, Nakao K, Morii N, Yamada T, Itoh H et al. (1988) Augmented synthesis of beta-human atrial natriuretic polypeptide in human failing hearts. Biochem Biophys Res Commun 150: 60-67.
4. [Rizzi C](http://www.ncbi.nlm.nih.gov/pubmed?term="Rizzi C"%5BAuthor%5D), Rossini K, Bruson A, Sandri M, Dal Belin Peruffo A et al. ( 2002) Fully reversible procedure for silver staining improves densitometry of complex mixtures of biopolymers resolved by sodium dodecyl sulfate-polyacrylamide gel electrophoresis. Electrophoresis 23: 3266-3269.
5. Levine H 3rd (1995) Soluble multimeric Alzheimer beta(1-40) pre-amyloid complexes in dilute solution. Neurobiol Aging 16: 755-764
6. [Craig WS](http://www.ncbi.nlm.nih.gov/pubmed?term="Craig WS"%5BAuthor%5D) (1988) Determination of quaternary structure of an active enzyme using chemical cross-linking with glutaraldehyde. Methods Enzymol 156: 333-345.
7. [Hermann R](http://www.ncbi.nlm.nih.gov/pubmed?term="Hermann R"%5BAuthor%5D), [Jaenicke R](http://www.ncbi.nlm.nih.gov/pubmed?term="Jaenicke R"%5BAuthor%5D), [Rudolph R](http://www.ncbi.nlm.nih.gov/pubmed?term="Rudolph R"%5BAuthor%5D) (1981) Analysis of the reconstitution of oligomeric enzymes by cross-linking with glutaraldehyde: kinetics of reassociation of lactic dehydrogenase. Biochemistry 20: 5195-5201.
8. Schägger H, von Jagow G (1987) Tricine-sodium dodecyl sulfate-polyacrylamide gel electrophoresis for the separation of proteins in the range from 1 to 100 kDa. Anal Biochem 166: 368-379.
9. Klunk WE, Pettegrew JW, Abraham DJ (1989) Quantitative evaluation of congo red binding to amyloid-like proteins with a beta-pleated sheet conformation. J Histochem Cytochem 37: 1273-1281.
10. LeVine H 3^rd^ (1999) Quantification of beta-sheet amyloid fibril structures with thioflavin T. Methods Enzymol 309: 274-284.
11. [Hong DP](http://www.ncbi.nlm.nih.gov/pubmed?term="Hong DP"%5BAuthor%5D), Fink AL (2005) Independent heterologous fibrillation of insulin and its B-chain peptide. Biochemistry 44: 16701-16709.
12. Timasheff SN (1992) Water as ligand: preferential binding and exclusion of denaturants in protein unfolding. Biochemistry 31: 9857-9864.
13. Bradford MM (1976) A rapid and sensitive method for the quantitation of microgram quantities of protein utilizing the principle of protein-dye binding. Anal Biochem 72: 248-254.
14. Fairbrother WJ, McDowell RS, Cunningham BC (1994) **Solution conformation of an atrial natriuretic peptide variant selective for the type A receptor.** Biochemistry 33: 8897-8904.
